# A Critical Time Window of Neocortex Modulation for Remote Fear Memory in Rats and Humans

**DOI:** 10.1002/advs.202520707

**Published:** 2026-09-30

**Authors:** Lu Cao, Yu‐Xin Zhang, Xiao‐Xing Liu, Zi‐Hang Li, Tang‐Sheng Lu, Jia‐Hui Deng, Wen Zhang, Peng Li, Xiao Lin, Shu‐Fei Feng, Ting‐Ting Li, Shi‐Qiu Meng, Xiang‐Dong Tang, Tian‐Ye Jia, Trevor W. Robbins, Jie Shi, Ying Han, Yan‐Xue Xue, Lin Lu, Kai Yuan

**Affiliations:** ^1^ Peking University Sixth Hospital Peking University Institute of Mental Health NHC Key Laboratory of Mental Health (Peking University) National Clinical Research Center for Mental Disorders (Peking University Sixth Hospital) Beijing People's Republic of China; ^2^ Shandong Institute of Brain Science and Brain‐inspired Research Medical Science and Technology Innovation Center Shandong First Medical University & Shandong Academy of Medical Sciences Jinan People's Republic of China; ^3^ National Institute on Drug Dependence and Beijing Key Laboratory of Drug Dependence Research Peking University Beijing People's Republic of China; ^4^ Department of Neurobiology School of Basic Medical Sciences Peking University Health Science Center Beijing People's Republic of China; ^5^ Sleep Medicine Center Department of Respiratory and Critical Care Medicine Mental Health Center Translational Neuroscience Center State Key Laboratory of Biotherapy West China Hospital Sichuan University Chengdu People's Republic of China; ^6^ Institute of Science and Technology for Brain‐Inspired Intelligence Fudan University Shanghai People's Republic of China; ^7^ Key Laboratory of Computational Neuroscience and Brain‐Inspired Intelligence (Fudan University) Ministry of Education Shanghai People's Republic of China; ^8^ Behavioural and Clinical Neuroscience Institute Department of Psychology University of Cambridge Cambridge UK; ^9^ Peking‐Tsinghua Center for Life Sciences and PKU‐IDG/McGovern Institute for Brain Research Peking University Beijing People's Republic of China

**Keywords:** fear memory, mPFC, remote memory, systems consolidation, VTA

## Abstract

Persistent fear memories, central to trauma‐related psychopathology, rely on long‐term consolidation. While initial encoding involves subcortical circuits, the precise timing and necessity of neocortical engagement for remote fear memory expression remain elusive. In the present translational study from rats to humans, we identified a late consolidation phase in the medial prefrontal cortex (mPFC) that occurs around 18 h after fear conditioning, a phase essential for remote memory. Neuronal ensembles in the mPFC activated during this phase mediate the expression of remote fear memory in rats. Critically, dopaminergic signaling in the mPFC modulated by ventral tegmental area (VTA) projections specifically during this delayed phase is required for remote fear memory maintenance. Consistent with these findings in humans, non‐invasive stimulation of homologous mPFC regions during this temporal window selectively reduced remote fear memory retrieval without affecting recent memory. These findings reveal an evolutionarily conserved temporal window for remote fear memory and identify novel time‐specific targets for clinical intervention in fear‐ and trauma‐related disorders.

## Graphical abstract

This study identifies a delayed consolidation window around 18 h after fear learning in the mPFC essential for remote fear memory. VTA dopaminergic input during this window sustains remote memory in rats, while non‐invasive stimulation of homologous human mPFC at the corresponding time selectively weakens remote but not recent fear memory, revealing a conserved time‐specific intervention target.

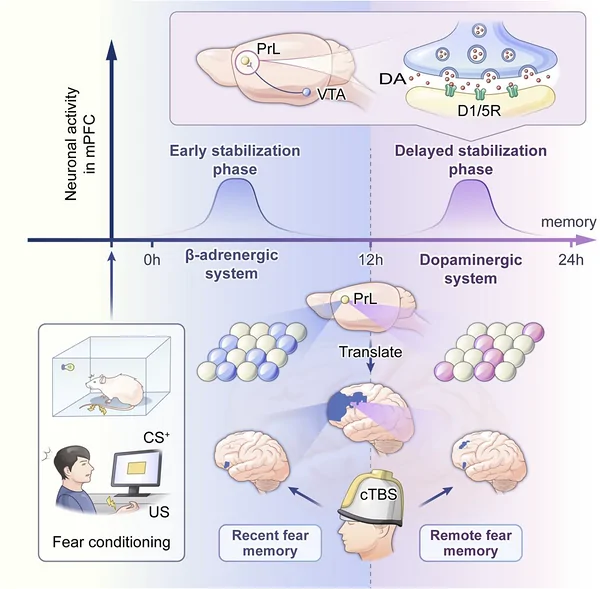

## 1 Introduction

The overgeneralization or dysregulation of fear memory following traumatic experiences gives rise to posttraumatic stress disorder—a key example of human fear disorders—as well as other fear‐ and trauma‐related disorders. These maladaptive memories, characterized by their profound emotional foundations, are extraordinarily robust and difficult to treat, particularly when they manifest after a prolonged delay [1, 2]. Previous findings indicate that attenuation of long‐lasting (remote) fearful memories is feasible through retrieval‐induced reconsolidation updating procedures in experimental settings [3, 4]. However, the clinical challenge remains significant—retrieval of remote fear memories in patients with fear‐ and trauma‐related disorders inevitably reactivates distressing experiences, underscoring the urgent need for more effective interventions with reduced adverse effects.

Systems consolidation theory emphasizes that newly formed episodic memories are initially encoded in the hippocampus and gradually transferred to distributed neocortical networks for long‐term storage [5, 6]. Within the hippocampus, successive waves of synaptic plasticity and molecular signaling organize engram cells and their wiring for persistent storage of memories [7, 8, 9]. The formation of remote fear memory involves complex interactions between the hippocampus and cortical regions, with the medial prefrontal cortex (mPFC) playing a particularly crucial role [10, 11, 12]. It has been suggested that neuronal ensembles in the mPFC supporting remote memory form rapidly during initial learning but undergo a transition from a silent to an unsilenced state throughout consolidation [13]. In addition, evidence has also indicated that distinct engram subpopulations (sub‐engrams) contribute to memory recall in recent memory vs. remote memory, with sub‐engrams' contribution determined by their projection profile [14]. However, the precise timing and necessity of neocortical involvement, as well as the specificity with which distinct molecular and cellular signatures are recruited for remote fear memory storage, remain largely elusive. Furthermore, it remains unclear whether cortical memory traces emerge merely through passive, incremental strengthening over time, or whether active consolidation processes occur at specific post‐learning time windows and thereby contribute to the stabilization of remote memories.

To address these questions, we employed complementary approaches in both rodents and humans. In rodents, combining activity‐dependent cell‐labeling, viral circuit‐specific manipulations, pharmacological and chemogenetic interventions, and other complementary techniques, we identified a delayed consolidation phase in the mPFC shortly after learning that is crucial for the persistence of remote memory. To assess translational relevance, we also conducted human studies using resting‐state functional magnetic resonance imaging (fMRI) and deep transcranial magnetic stimulation (dTMS) targeting the mPFC during this delayed phase. This cross‐species experimental design allowed us to evaluate the evolutionary conservation of prefrontal late‐stage consolidation mechanisms and their potential therapeutic applications.

## 2 Results

### 2.1 Delayed Engagement of the mPFC is Critical for the Expression of Remote Memory in Rats

Expression of the immediate‐early gene c‐*Fos* and the protein c‐Fos has been extensively used as a marker of neural activity [15]. To map the temporal dynamics of mPFC activity following memory acquisition, we examined c‐Fos protein levels at different time points following contextual fear conditioning (CFC), in which the rats learn to associate a neutral context with aversive unconditioned stimuli (US; electric footshock) and express fear responses to the context. We found two waves of c‐Fos expression in the prelimbic cortex (PrL): an early wave detected at 0.5–1.5 h post‐training (Figure S1) and a delayed wave emerging at approximately 18 h after contextual fear memory training, with the majority of c‐Fos^+^ cells located in layers 5b–6 but not layers 2–5a during the delayed phase (Figure 1A–C and Figure S2A–C). In contrast, there was only an early wave of c‐Fos expression in the infralimbic cortex (IL), another subregion of the mPFC (Figure S2D, E). Furthermore, the neurons recruited during the early and delayed waves of c‐Fos expression were largely excitatory neurons (Figure S3). No statistically significant difference in c‐Fos expression relative to naive controls was detected across the 3, 6, 9, 12, 24, and 36 h time points following contextual fear memory training (Figure 1B,C). Moreover, Western blot and in situ hybridization results showed that c‐Fos expression was also significantly increased during both early and delayed phases (Figures S4 and S5). To further confirm the increased activity of the PrL during the early and delayed phases, we evaluated spontaneous excitatory postsynaptic currents (sEPSCs) in the PrL pyramidal neurons (Figure 1D–G). The results demonstrated that, compared with the naive control group, sEPSC frequency was significantly increased during both the early (0.5 h post‐training) and delayed (18 h post‐training) phases (Figure 1F). Consistent with these findings, sEPSC amplitude was also significantly elevated during the two phases (Figure 1G). These results collectively indicate enhanced spontaneous synaptic transmission in the PrL pyramidal neurons during these two critical time windows of mPFC activity.

**FIGURE 1:**
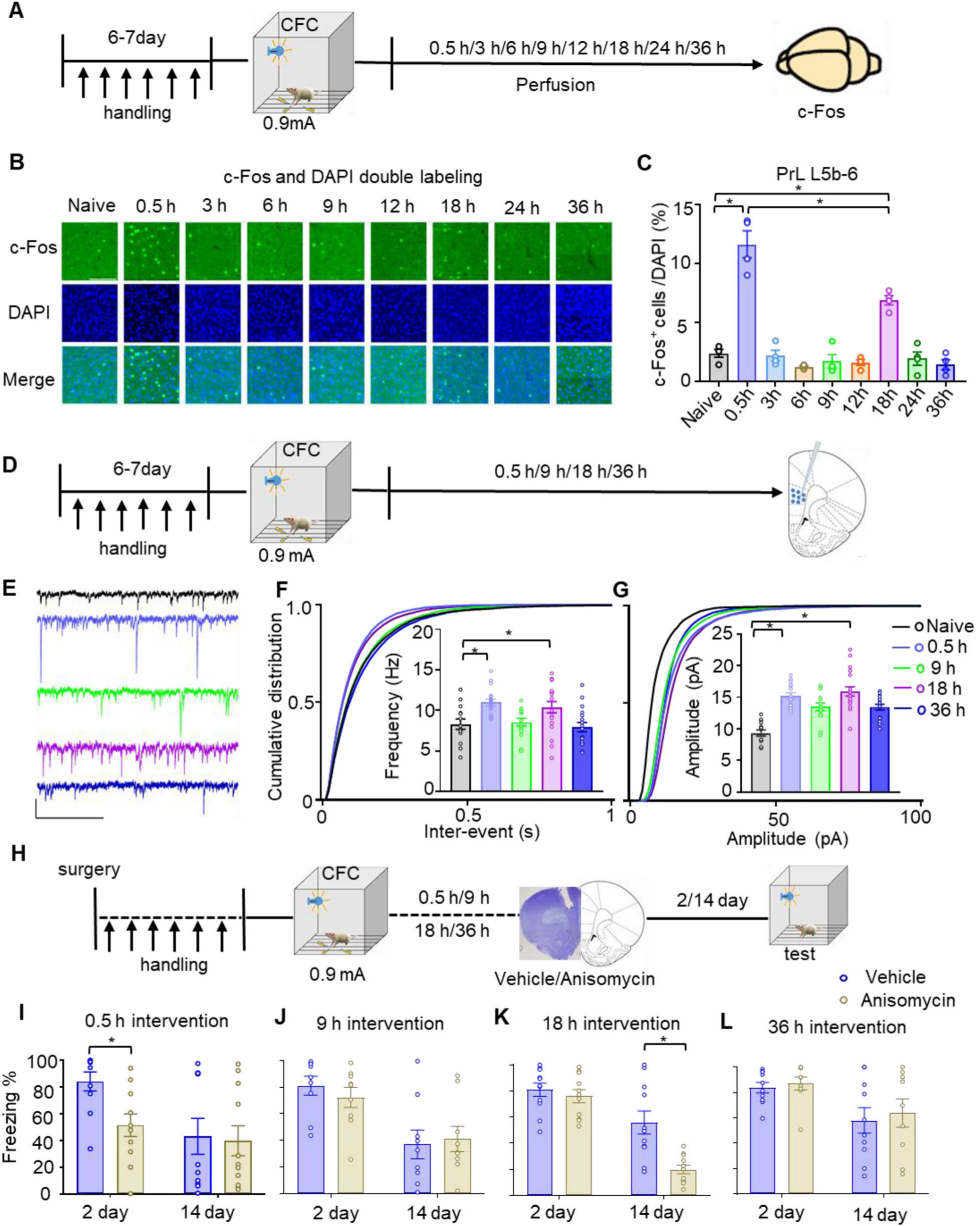
Delayed activation of the mPFC is required for the expression of remote fear memory. (A) Timeline. After contextual fear conditioning (CFC), the rats were perfused at 0.5, 3, 6, 9, 12, 18, 24, and 36 h, and the number of c‐Fos^+^ cells in the PrL was detected by immunofluorescence. (B) Representative images of c‐Fos and DAPI immunostaining in the PrL. Scale bar = 100 µm. (C) Percentage of c‐Fos^+^ cells per DAPI^+^ cells in layers 5b‐6 (one‐way ANOVA followed by Bonferroni‐corrected post hoc tests, *F*_(8, 27)_ = 41.70, *p* < 0.01 vs. Naive). The data are expressed as the mean ± SEM. *n* = 4/group. (D) Timeline of sEPSC recordings of PrL pyramidal neurons 0.5, 9, 18, and 36 h after contextual fear memory training. (E) Example sEPSC traces from PrL pyramidal neurons in the naive, 0.5, 9, 18, and 36 h groups after contextual fear memory training. Scale bar: 20 pA (vertical), 1 s (horizontal). (F) Cumulative distribution of sEPSC frequency and dot plots (inset) that show that PrL pyramidal neurons in the 0.5 and 18 h groups had significantly higher sEPSC frequencies compared with PrL pyramidal neurons in the naive group (one‐way ANOVA followed by Bonferroni‐corrected post hoc tests, *F_(_*_4, 77)_ = 5.70, *p* < 0.001; a priori planned comparisons using unpaired t‐tests, 0.5 h vs. Naive, *p <* 0.005; 18 h vs. Naive, *p* = 0.041). (G) Cumulative distribution of sEPSC amplitude and dot plots (inset) that show that PrL pyramidal neurons in the 0.5 and 18 h groups had significantly higher sEPSC amplitudes compared with PrL pyramidal neurons in the naive group (one‐way ANOVA followed by Bonferroni‐corrected post hoc tests, *F_(_*_4, 77)_ = 18.46, *p* < 0.001; a priori planned comparisons using unpaired t‐tests, 0.5 h vs. Naive, *p* < 0.001; 18 h vs. Naive, *p* < 0.001). The data in F and G are expressed as the mean ± SEM. ^*^ *p* < 0.05. *n* = 14–19 neurons and 7–9 sections from 3 rats per group. Frequencies in the inset are reported in Hz for clarity, derived from inter‐event intervals. (H) Timeline of animals that were infused with vehicle or anisomycin into the PrL 0.5, 9, 18, or 36 h after contextual fear memory training. (I) Recent but not remote fear memory decreased after anisomycin was injected into the PrL 0.5 h after contextual fear memory training (drug × test day interaction: *F*_(1, 19)_ = 4.54, *p* = 0.046). (J, L) The microinjection of anisomycin into the PrL 9 and 36 h after contextual fear memory training had no effect on the expression of recent or remote fear memory (*p* > 0.05). (K) Remote but not recent memory decreased after anisomycin was injected into the PrL 18 h after contextual fear memory training (drug × test day interaction: *F*_(1, 19)_ = 9.81, *p* = 0.005). The data in I–L are expressed as the mean ± SEM. ^*^ *p* ≤ 0.05 (Two‐way repeated‐measures ANOVA followed by Bonferroni‐corrected post hoc tests). *n* = 8–12 rats/group.

To test whether *de novo* protein synthesis, a hallmark of synaptic and systems consolidation [16], in the PrL during the early and delayed phases is required for the persistence of fear memory, we injected the protein synthesis inhibitor anisomycin (Figure 1H). Rats were trained in a contextual fear conditioning procedure and then tested on Day 2 (recent memory) and Day 14 (remote memory). Since anisomycin exerts an inhibitory effect on protein synthesis that lasts around 5 h [17], we selected 0.5 h post‐training as the time point of early intervention to cover the entire early activation window. The infusion of anisomycin in the PrL at 0.5 h post‐training significantly decreased fear memory recall in the Day 2 test but not in the Day 14 test (Figure 1I). Notably, the infusion of anisomycin in the PrL at 18 h significantly decreased fear memory recall in the Day 14 test but not in the Day 2 test (Figure 1K). In addition, microinfusion of anisomycin at 0.5 h after contextual fear memory training did not affect c‐Fos expression at 18 h (Figure S6). No changes in memory recall were observed when anisomycin was infused at 9 or 36 h after training compared with the vehicle group (Figure 1J,L). These results indicated that there were also two waves of protein synthesis in the PrL after fear memory learning. The early wave is required for the retrieval of recent memory, while the delayed wave is necessary for the retrieval of remote memory.

We further replicated these findings with another protein synthesis inhibitor, rapamycin, which acts on mammalian/mechanistic target of rapamycin activity, inhibiting its downstream signaling and modulating translation [18]. The infusion of rapamycin in the PrL at 18 h but not 0.5 h significantly decreased fear memory recall on Day 14 but not on Day 2 (Figure S7), indicating that mTOR‐dependent protein synthesis is required during the delayed phase. To investigate whether the hippocampus‐independent auditory fear memory also undergoes protein synthesis‐dependent delayed activation in the PrL, we used an auditory fear conditioning (AFC) procedure, during which animals learn the association between a discrete (auditory) cue and the US and display fear to the cue, to further validate our findings (Figure S8A). Rats that received anisomycin at 18 h froze significantly less than control rats on the Day 14 test but not on the Day 2 test (Figure S8D, E). No changes in freezing behavior were observed in rats that received anisomycin at 0.5 h and the vehicle group on the Day 2 test or the Day 14 test (Figure S8B, C). These results suggest that the delayed protein‐synthesis‐dependent engagement of the PrL is critical for the expression of remote memory across the hippocampus‐dependent and ‐independent fear memories.

### 2.2 Delayed Recruitment of Neuronal Ensembles in the mPFC is Required for Remote Fear Memory Retrieval

Recent evidence has suggested a critical role of neuronal ensembles, the sparsely distributed neurons that encode memory traces, in fear memory formation and consolidation [5, 13]. We therefore investigated the neuronal populations activated during the early and delayed phases of mPFC activity. We first labeled neuronal populations at approximately 0.5 h post‐training using either a tet‐OFF lentiviral vector expressing enhanced green fluorescent protein (EGFP; Figure 2A) or a tamoxifen‐inducible Cre‐loxP recombination system (Figure S9A) [13], then co‐localized these tagged neurons with endogenous c‐Fos expression at 18 h post‐training (Figure 2B and Figure S9B). In the home cage control group without associative learning, few neurons were labeled or expressed c‐Fos (Figure 2C and Figure S9C). In contrast, the CFC group exhibited significantly more early‐labeled (neurons activated during CFC training and around 0.5 h post‐training) and c‐Fos‐positive (neurons activated at 18 h post‐training) neurons relative to controls (Figure 2C and Figure S9C). Importantly, minimal overlap was detected between early‐labeled and c‐Fos‐positive neurons in both groups. The proportion of double‐labeled neurons did not differ from the predicted chance level, calculated as the product of the fractions of each single‐labeled population (observed: [EGFP^+^ c‐Fos^+^]/DAPI^+^ × 100 vs. chance: [EGFP^+^/DAPI^+^]×[c‐Fos^+^/DAPI^+^] × 100 (Figure 2C); observed: [mCherry^+^ c‐Fos^+^]/DAPI^+^ × 100 vs. chance: [mCherry^+^/DAPI^+^]×[c‐Fos^+^/DAPI^+^] × 100 (Figure S9C)). Together, these findings indicate that neurons activated during early and delayed phases belong to different neuronal ensembles.

**FIGURE 2:**
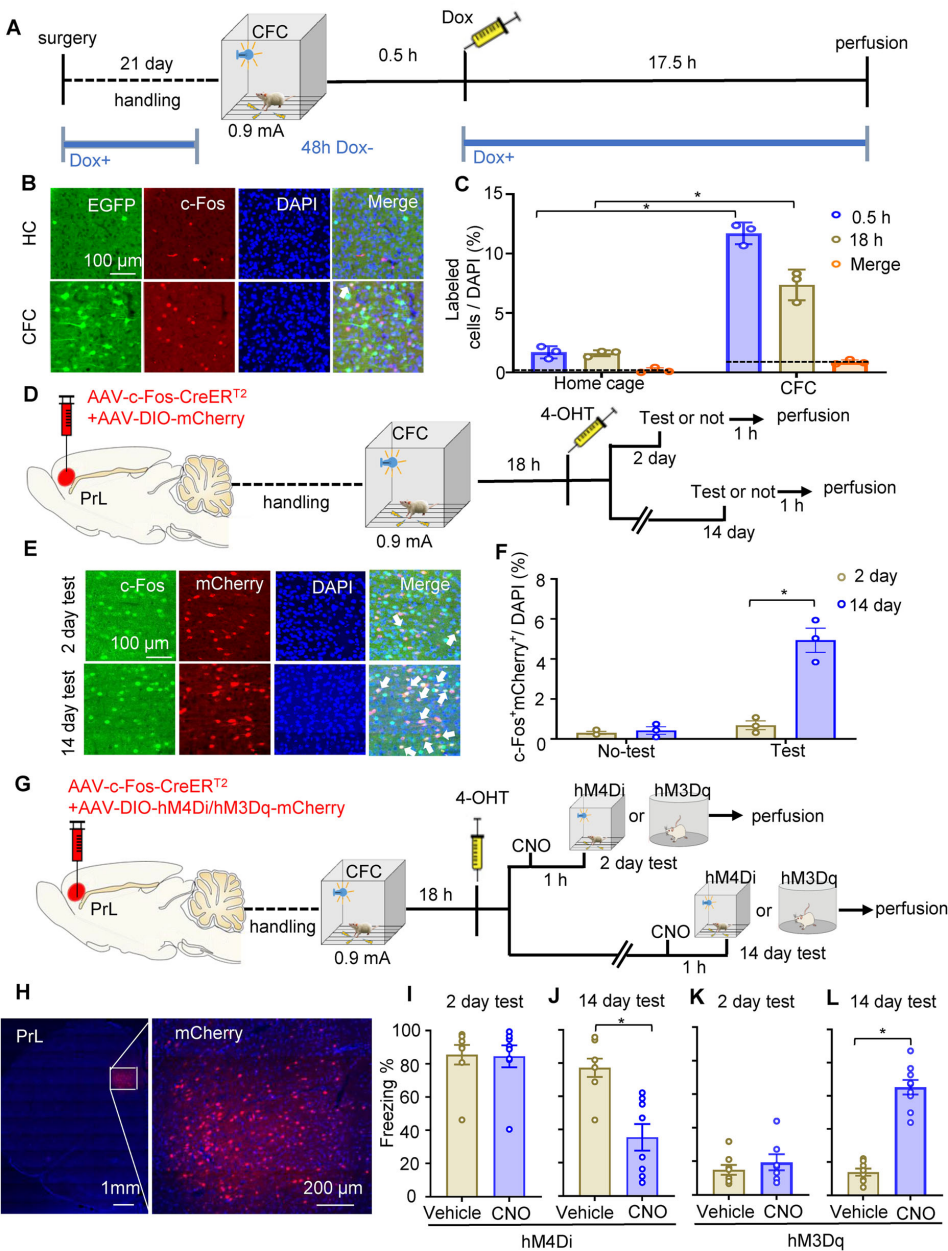
Neuronal ensembles recruited during the delayed activation phase of the mPFC drive remote fear memory expression. (A) Experimental timeline for labeling mPFC neurons at approximately 0.5 h and immunofluorescence staining for endogenous c‐Fos expression at 18 h following CFC. (B) Representative fluorescence microscopy images showing tagged neurons in the prelimbic cortex (PrL). Neurons activated in the home cage were labeled in the home cage group, whereas early‐activated neurons were labeled in the CFC group, and delayed‐activated neurons were identified by c‐Fos immunolabeling. Scale bar = 100 µm. (C) Percentage of EGFP^+^ cells or c‐Fos^+^ cells relative to total DAPI^+^ cells in the PrL (CFC vs. home cage, unpaired t‐test, *p* < 0.005); The proportion of EGFP^+^c‐Fos^+^/DAPI^+^ neurons did not differ significantly from the expected chance level, calculated as the product of the fractions of EGFP^+^/DAPI^+^ × c‐Fos^+^/DAPI^+^ neurons in both the home cage and the CFC groups. *n* = 3 rats per group. (D) Experimental timeline for activity‐dependent labeling and memory tests. Animals received an intraperitoneal injection of 4‐OHT 18 h post‐CFC, followed by memory tests performed at either 2 or 14 days post‐conditioning. Brain tissue was collected 60 min after behavioral tests for c‐Fos immunohistochemistry. (E) Representative immunofluorescence images of the PrL region showing labeled neurons. Scale bar = 100 µm. (F) Quantification of double‐labeled cells (c‐Fos^+^ and mCherry^+^) as a percentage of total DAPI^+^ cells in the PrL (*F*_(1, 4)_ = 30.20*, p* = 0.005), *n* = 3 rats per group. (G) Experimental timeline for chemogenetic manipulation. 4‐OHT was administered intraperitoneally 18 h post‐CFC to enable c‐Fos‐driven hM4Di/hM3Dq expression. CNO was administered 60 min prior to memory testing. (H) Representative images showing viral expression in the PrL. (I–L) Freezing behavior during recent and remote memory tests (The data in I–L are expressed as the mean ± SEM. *n* = 7–9 rats/group. ^*^ *p* ≤ 0.05, unpaired t‐test).

Combined with the preceding data, we speculate that distinct neuronal ensembles underlying early‐ and delayed‐ phase activation support the expression of recent and remote fear memories, respectively. Therefore, we next investigated whether neuronal ensembles activated during the delayed phase are re‐engaged during remote fear memory retrieval. We labeled these neurons with mCherry via 4‐OHT injection 18 h after contextual fear conditioning, and identified cells activated during memory tests using c‐Fos immunostaining (Figure 2D,E). In the absence of memory tests, c‐Fos expression in mCherry‐positive neurons within the PrL did not differ significantly between Day 2 and Day 14 (Figure 2F). Importantly, only memory testing performed on Day 14, but not on Day 2, significantly increased c‐Fos expression in mCherry‐positive cells, as assessed by the proportion of double‐labeled (mCherry^+^/c‐Fos^+^) cells. (Figure 2F). This suggests that the delayed neuronal ensembles are critical to remote fear memory retrieval.

To establish the necessity of delayed ensembles in remote memory retrieval, we bilaterally injected AAV9‐c‐Fos‐ERT2CreERT2 and AAV9‐hSyn‐DIO‐hM4Di‐mCherry into the PrL. Neurons activated during the delayed phase were labeled with hM4Di‐mCherry through 4‐OHT injection 18 h post‐CFC, and were subsequently inhibited during memory tests via clozapine‐*N*‐oxide (CNO; a DREADD agonist) administration [19] (Figure 2G, H). Inhibition of these neuronal ensembles significantly reduced freezing behavior when tested on Day 14 but not on Day 2 (Figure 2I,J). To further investigate the sufficiency of delayed ensembles in remote memory retrieval, we bilaterally injected AAV9‐c‐Fos‐ERT2CreERT2 and AAV9‐hSyn‐DIO‐hM3Dq‐mCherry into the PrL. Neurons activated during the delayed phase were labeled with hM3Dq‐mCherry through 4‐OHT injection 18 h post‐CFC, and were subsequently activated during memory tests in a novel context distinct from the training environment via CNO administration [19] (Figure 2G,H). Activating these neuronal ensembles significantly increased freezing behavior when tested on Day 14 in the other context but not on Day 2 (Figure 2K,L). Moreover, optogenetic stimulation at 4 Hz, but not 20 Hz, of ChR2‐mCherry–expressing neurons in the delayed activation phase of the PrL induced increased freezing behavior on Day 14 but not Day 2 in an unconditioned context (Figure S10C). These results demonstrate that the delayed‐activated neuronal ensembles are critical for remote fear memory retrieval.

### 2.3 Dopaminergic Signaling During the Delayed Phase of mPFC Mediates Remote Fear Memory Expression

We next investigated the specific modulatory mechanisms of the early and delayed phases of the PrL, which contribute to the retrieval of recent and remote memory, respectively. Previous studies have suggested that β‐adrenergic receptors [20] and dopamine D_1_/D_5_ receptors in the hippocampus play a crucial role in the formation and persistence of long‐term fear memory [7]. We therefore investigated whether these two neuromodulatory systems participate in the two phases. We first bilaterally infused rats with dopamine D_1_/D_5_ receptor antagonist SCH23390 or agonist SKF38393 0.5 or 18 h after contextual fear memory training (Figure 3A). The SCH23390 injection in the PrL at 18 h specifically disrupted retrieval of memory on Day 14 but not on Day 2 (Figure 3B), while the SKF38393 injection at 18 h enhanced remote memory recall but not recent recall (Figure 3C). The effect of SKF38393 was not due to memory generalization, as freezing behavior in another context on Day 15 showed no difference between the two groups (Figure S11A, B). SCH23390 and SKF38393 had no effect on the retrieval of fear memory on Day 2 or Day 14 when given 0.5 h after training (Figure S12A–C). However, the injection of β‐adrenergic receptor antagonist propranolol at 0.5 h blocked memory retrieval in the Day 2 test but not in the Day 14 test (Figure S12A, D), while the propranolol injection at 18 h had no effect on either recent or remote recall (Figure 3D). These results indicate that dopaminergic signaling, but not noradrenergic signaling, during the delayed phase of the PrL is critical for the retrieval of remote fear memory.

**FIGURE 3:**
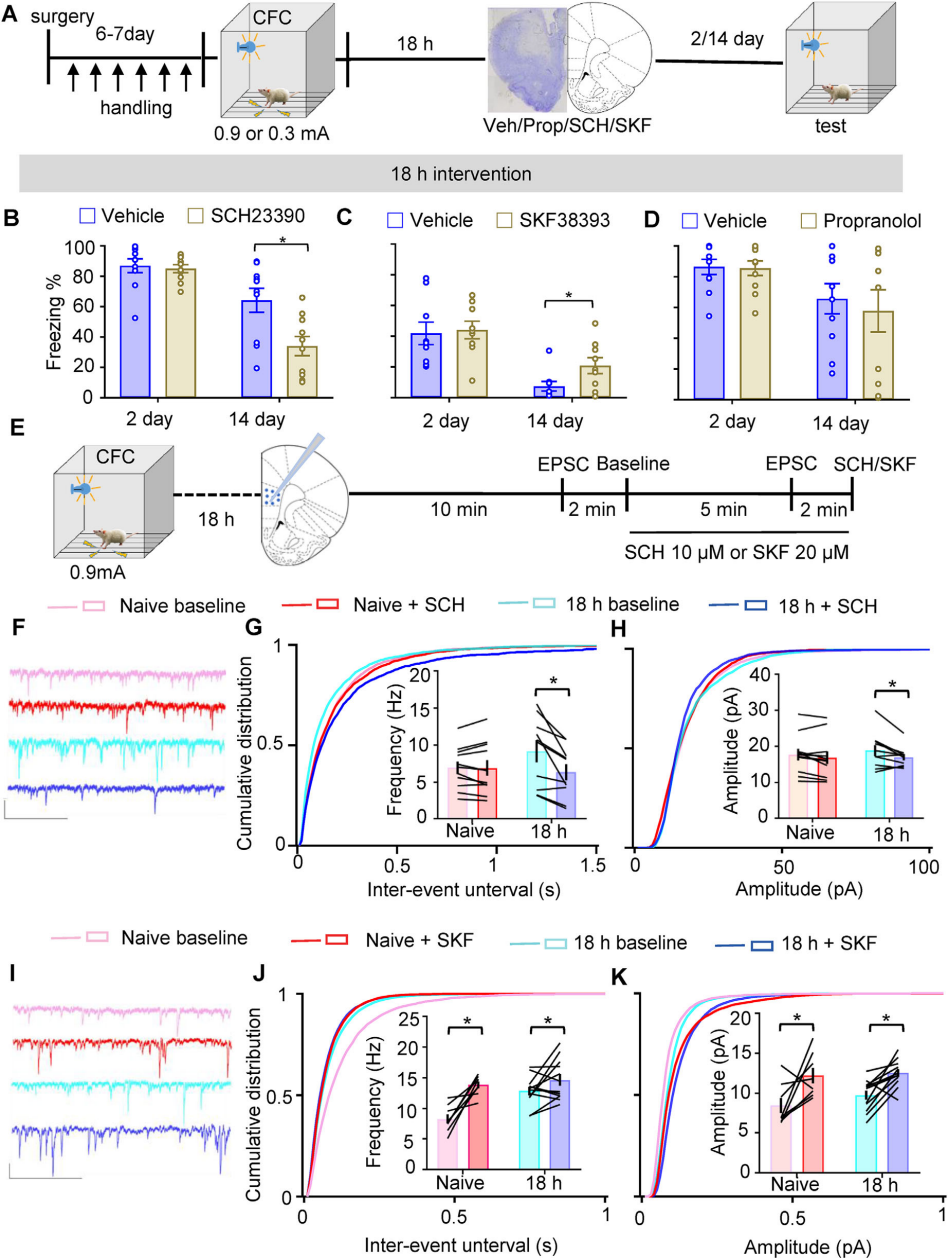
Dopamine D1/D5 receptors modulate delayed mPFC actions on remote fear memory. (A) Rats were infused with vehicle or SCH23390/SKF38393/propranolol into the PrL 18 h after contextual fear memory training. (B) Remote but not recent memory decreased after SCH23390 was injected into the PrL 18 h after contextual fear memory training (drug × test day interaction: *F*_(1, 18)_ = 5.03*, p* = 0.038). (C) The microinjection of SKF38393 into the PrL 18 h after contextual fear memory training promoted the maintenance of remote fear memory (unpaired t‐test, *p* = 0.041). (D) The microinjection of propranolol into the PrL 18 h after contextual fear memory training had no effect on the expression of recent or remote fear memory (*p* > 0.05). The data in B‐D are expressed as the mean ± SEM. ^*^ *p* ≤ 0.05. *n* = 8–12 rats/group. We used weak training (0.3 mA) for the agonist SKF38393 experiment to provide headroom for enhancement effects, and normal training (0.9 mA) for antagonist experiments. (E) Timeline of sEPSC recordings of PrL pyramidal neurons before and after administration of the D_1_/D_5_ receptor antagonist SCH23390 or the D_1_/D_5_ receptor agonist SKF38393 in the naive group and 18 h group after contextual fear memory training. (F) Example sEPSC traces from PrL pyramidal neurons in the naive group (pink: naive baseline; red: naive SCH23390) and 18 h group (light blue: 18 h baseline; dark blue: 18 h SCH23390). Scale bar: 20 pA (vertical), 1 s(horizontal). (G) Cumulative distribution of sEPSC inter‐event intervals and dot plots (inset) showed that PrL pyramidal neurons in the 18 h group had significantly lower sEPSC frequencies after SCH23390 administration (*F*_(1, 19)_ = 12.48, *p* = 0.0022). (H) Cumulative distribution of sEPSC amplitudes and dot plots (inset) showed that PrL pyramidal neurons in the 18 h group had lower sEPSC amplitudes after SCH23390 administration (18 h + SCH vs. 18 h baseline: paired t‐test, *p* = 0.042). (I) Example sEPSC traces from PrL pyramidal neurons in the naive group (pink: naive baseline; red: naive SKF38393) and 18 h group (light blue: 18 h baseline; dark blue: 18 h SKF38393). Scale bar: 20 pA (vertical), 1 s(horizontal). (J) Cumulative distribution of sEPSC inter‐event intervals and dot plots (inset) showed that the PrL pyramidal neurons both in the naive group and the 18 h group had significantly higher sEPSC frequencies after SKF38393 administration (Naive + SKF vs. Naive baseline: paired t‐test, *p* < 0.001; 18 h + SKF vs. 18 h baseline: paired t‐test, *p* = 0.036). (K) Cumulative distribution of sEPSC amplitudes and dot plots (inset) showed that PrL pyramidal neurons in the naive group or 18 h group had significantly higher sEPSC amplitude after SKF38393 administration (Naive + SKF vs. Naive baseline: paired t‐test, *p* = 0.039; 18 h + SKF vs. 18 h baseline: paired t‐test, *p* = 0.0021). The data in G, H, J, and K are expressed as the mean ± SEM, ^*^ *p* ≤ 0.05, *n* = 8–10 neurons and 8–10 sections from 3 rats per group. Frequencies in the inset are reported in Hz for clarity, derived from inter‐event intervals.

To investigate how D_1_/D_5_ receptors modulate synaptic function in the PrL during the delayed phase, ex vivo electrophysiology experiments were performed to examine sEPSCs of PrL pyramidal neurons after contextual fear memory training. Results showed that SCH23390 blocked the increase in sEPSC frequency and amplitude during the delayed phase of PrL pyramidal neurons (Figure 3F–H). SCH23390 had no effect on either frequency or amplitude of sEPSCs of PrL pyramidal neurons in naive rats. In contrast, SKF38393 increased both the frequency and amplitude of sEPSCs in PrL pyramidal neurons from naive and 18 h groups (Figure 3I–K). In addition, propranolol blocked the increase in both sEPSC frequency and amplitude in the early phase (Figure S12E–H). These results indicate that D_1_/D_5_ receptors are involved in the enhancement of spontaneous synaptic transmission in the PrL pyramidal neurons during the delayed phase.

### 2.4 Dopamine D_1_/D_5_ Receptors in the PrL Modulated by VTA Projections Gate Remote Fear Memory Expression

To further identify the functional inputs to the PrL during the delayed phase, we injected Retro‐AAV‐EGFP into the PrL (Figure 4A). The results showed that neurons in the VTA expressed EGFP (Figure 4B), indicating an anatomical projection from the VTA to the PrL. Next, we investigated the necessity of the VTA‐PrL projection during the delayed phase in the persistence of remote memory. To specifically inhibit the VTA‐PrL projection, we injected Retro‐AAV‐Cre‐EGFP into the PrL and AAV‐DIO‐hM4Di‐mCherry (or AAV‐DIO‐mCherry as a control) into the VTA (Figure 4C–E). After 3 weeks, CNO was administered intraperitoneally (i.p.) 17 h after contextual fear memory training. Compared with the control groups, delayed inhibition of the VTA‐PrL projection had no effect on the retrieval of recent memory on Day 2 but specifically impaired the retrieval of remote memory on Day 14 (Figure 4F). As expected, this manipulation significantly decreased c‐Fos levels in the PrL compared with the control groups (Figure S13A,C).

**FIGURE 4:**
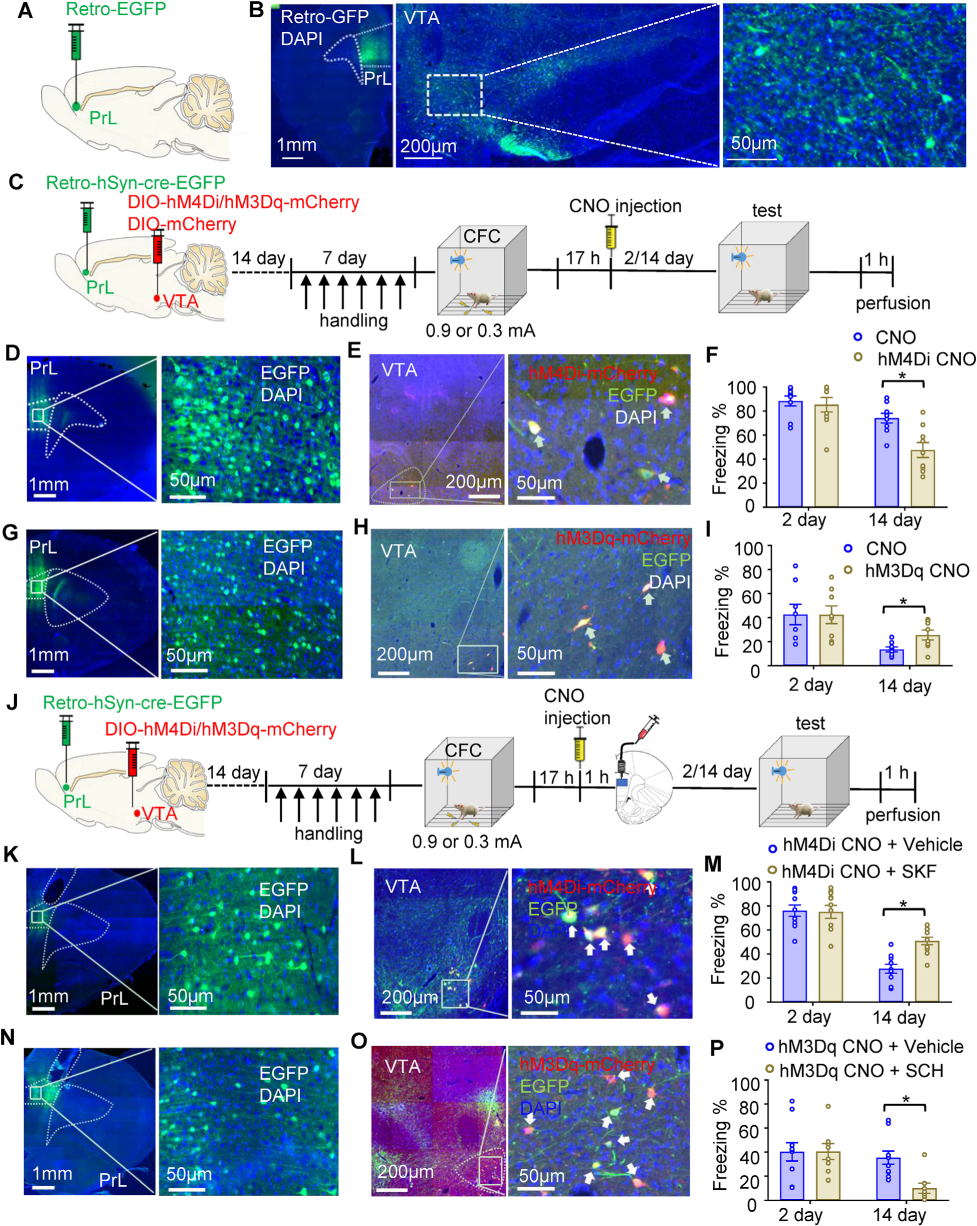
The delayed engagement of VTA‐mPFC projection modulates remote fear memory via dopamine D_1_/D_5_ receptors in the PrL. (A) Retro‐AAV‐hSyn‐EGFP‐WPRE‐hGH (Retro‐EGFP) in the PrL. (B) (Left) The image shows Retro‐EGFP and DAPI staining in the PrL (scale bar = 1 mm). (Middle) The image shows Retro‐EGFP and DAPI staining in the VTA (scale bar = 200 µm). (Right) Retro‐EGFP‐labeled neuron from the PrL co‐labeled with DAPI in the VTA (scale bar = 50 µm). (C) Schematic of the experiment. Retro‐AAV‐hSyn‐Cre‐EGFP was injected into the PrL, and rAAV‐EF1α‐DIO‐hM4Di‐mCherry/rAAV‐EF1α‐DIO‐hM3Dq‐mCherry/rAAV‐EF1α‐DIO‐mCherry was injected into the VTA. Three weeks later, the rats underwent contextual fear memory training, and CNO was injected 17 h after CFC. Brains were removed 60 min after the remote fear memory test. (D) (Left) Retro‐Cre‐EGFP expressed at the PrL injection site (scale bar = 1 mm). (Right) Local magnification of Retro‐Cre‐EGFP expressed in the PrL (green: Retro‐Cre‐EGFP; blue: DAPI; scale bar = 50 µm). (E) (Left) Retro‐Cre‐EGFP and DIO‐ hM4Di‐mCherry (DIO‐mCherry for control) expressed in the VTA. (Right) Local magnification of virus expression in the VTA (green: Retro‐Cre‐EGFP; red: DIO‐ hM4Di ‐mCherry or DIO‐mCherry; blue: DAPI; scale bar = 50 µm). (F) After contextual fear memory training, CNO application at 17 h significantly decreased remote fear memory but had no effect on recent fear memory (two‐way ANOVA followed by Bonferroni‐corrected post hoc tests, *F*_(1, 16)_ = 31.27, *p* < 0.001). *n* = 9 rats/group. (G) (Left) Retro‐EGFP‐Cre expressed at the PrL injection site (scale bar = 1 mm). (Right) Local magnification of Retro‐Cre‐EGFP expressed in the PrL (green: Retro‐Cre‐EGFP; blue: DAPI; scale bar = 50 µm). (H) (Left) Retro‐Cre‐EGFP and DIO‐ hM3Dq‐mCherry (DIO‐mCherry for control) expressed in the VTA. (Right) Local magnification of virus expression in the VTA (green: Retro‐Cre‐EGFP; red: DIO‐ hM3Dq‐mCherry or DIO‐mCherry; blue: DAPI; scale bar = 50 µm). (I) After contextual fear memory training, CNO application at 17 h promoted the maintenance of remote fear memory (unpaired t‐test, *p* < 0.05). *n* = 8 rats/group. (J) Schematic of the experiment. Retro‐Cre‐EGFP was injected into the PrL, and DIO‐ hM4Di‐mCherry/DIO‐hM3Dq‐mCherry was injected into the VTA. Three weeks later, the rats received a CNO injection 17 h after contextual fear memory training, and vehicle or SKF38393/SCH23390 was injected into the PrL 1 h later. (K) (Left) Retro‐Cre‐EGFP expressed at the PrL injection site (scale bar = 1 mm). (Right) Local magnification of Retro‐Cre‐EGFP expressed in the PrL (green: Retro‐Cre‐EGFP; blue: DAPI; scale bar = 50 µm). (L) (Left) Retro‐Cre‐EGFP and DIO‐ hM4Di‐mCherry expressed in the VTA. (Right) Local magnification of virus expression in the VTA (green: Retro‐Cre‐EGFP; red: DIO‐ hM4Di‐mCherry; blue: DAPI; scale bar = 50 µm). (M) Chemogenetic inhibition of the VTA‐PrL projection impaired the expression of remote fear memory but not in rats that received SKF38393 in the PrL (two‐way ANOVA followed by Bonferroni‐corrected post hoc tests, *F*_(1, 19)_ = 9.71, *p* = 0.006). *n* = 10–11 rats/group. (N) (Left) Retro‐Cre‐EGFP expressed at the PrL injection site (scale bar = 1 mm). (Right) Local magnification of Retro‐Cre‐EGFP expressed in the PrL (green: Retro‐Cre‐EGFP; blue: DAPI; scale bar = 50 µm). (O) (Left) Retro‐Cre‐EGFP and DIO‐hM3Dq‐mCherry expressed in the VTA. (Right) Local magnification of virus expression in the VTA (green: Retro‐Cre‐EGFP; red: DIO‐hM3Dq‐mCherry; blue: DAPI; scale bar = 50 µm). (P) Chemogenetic activation of the VTA‐PrL projection promoted the expression of remote fear memory but not in rats that received SCH23390 in the PrL (unpaired t‐test, *p* = 0.003). *n* = 8–10 rats/group. The data in F, I, M, and P are expressed as the mean ± SEM. ^*^ *p* ≤ 0.05.

Next, we activated the VTA‐PrL projection at 18 h and assessed the expression of remote memory. We injected Retro‐AAV‐Cre‐EGFP into the PrL and AAV‐DIO‐hM3Dq‐mCherry (or AAV‐DIO‐mCherry as a control) into the VTA (Figure 4G, H). After 3 weeks, CNO was administered 17 h after weak contextual fear memory training. Compared with the control groups, delayed engagement of the VTA‐PrL projection had no effect on the retrieval of recent memory on Day 2 but significantly enhanced the retrieval of remote memory on Day 14 (Figure 4I). This effect was not due to memory generalization, as fear memory tested in another context on Day 15 showed no difference between the two groups (Figure S14A, B). As expected, this manipulation significantly increased c‐Fos levels in the PrL compared with the control groups (Figure S13B, D). These results indicate that the delayed VTA‐PrL projection is required for the expression of remote memory.

To investigate the role of the VTA‐PrL projection during the early phase, we injected Retro‐AAV‐Cre‐EGFP into the PrL and AAV‐DIO‐hM4Di‐mCherry/AAV‐DIO‐hM3Dq‐mCherry into the VTA (Figure S15A–C, E, F). CNO (i.p.) was administered immediately after contextual fear conditioning. Compared with the control groups, early inhibition or activation of the VTA‐PrL projection had no effect on memory retrieval at either Day 2 or Day 14 (Figure S15D, G).

To test whether dopamine D_1_/D_5_ receptors in the PrL are modulated by the delayed VTA input, we inhibited the VTA‐PrL projection at 18 h and simultaneously activated dopaminergic signaling by injecting the D_1_/D_5_ receptor agonist SKF38393 into the PrL to determine whether this manipulation rescued the impairments of remote memory recall. We injected Retro‐AAV‐Cre‐EGFP into the PrL and AAV‐DIO‐hM4Di‐mCherry/AAV‐DIO‐hM3Dq‐mCherry into the VTA (Figure 4J–L). CNO was administered intraperitoneally 17 h after contextual fear conditioning. One hour later, SKF38393 was injected in the PrL. The results showed that SKF38393 enhanced remote recall but not recent recall (Figure 4M). Similarly, we also activated the VTA‐PrL projection at 18 h and simultaneously inhibited dopaminergic signaling by injecting the D_1_/D_5_ receptor antagonist SCH23390 into the PrL to determine whether this manipulation reversed the enhancement of remote memory recall (Figure 4N,O). As expected, SCH23390 blocked the enhancement of remote recall induced by delayed activation of the VTA‐PrL projection (Figure 4P). Locomotor activity (total distance traveled or time spent in the center zone) was unchanged among these groups (Figure S16). Altogether, these results indicate that the delayed VTA‐PrL projection contributes to the retrieval of remote memory through modulating dopamine D_1_/D_5_ receptors in the PrL.

### 2.5 Continuous Theta‐Burst Stimulation Over mPFC During the Delayed Phase Attenuates Remote Fear Memory Expression in Humans

The above animal experiments suggest that the delayed engagement of mPFC is critical for the expression of remote memory. We next investigated whether similar mechanisms operate in humans. Previous studies have identified the mPFC in Brodmann Areas 24 and 32 (BA 24 and BA 32) as the homologous regions to the rat prelimbic cortex [21, 22]. We then applied the continuous theta‐burst stimulation (cTBS), a non‐invasive brain stimulation technique that can alter the efficiency of synaptic transmission and thereby change the process of learning and memory [23, 24], to BA 24 and BA 32 regions during different time windows and assessed fear memory expression in humans (Figure 5A). Notably, evidence has suggested that memory consolidation and reconsolidation occur across comparable timescales in both animal and human studies [25, 26], potentially reflecting conserved molecular and cellular processes (e.g., protein synthesis and synaptic plasticity) underlying memory formation across species. Accordingly, in our human experiments, we defined approximately 0.5 h after learning as the early phase and approximately 18 h after learning as the delayed phase. Participants were randomly assigned to four temporal groups: 0.5, 9, 18, and 36 h post‐acquisition. Within each group, participants were randomly assigned (1:1) to receive either cTBS or sham stimulation.

**FIGURE 5:**
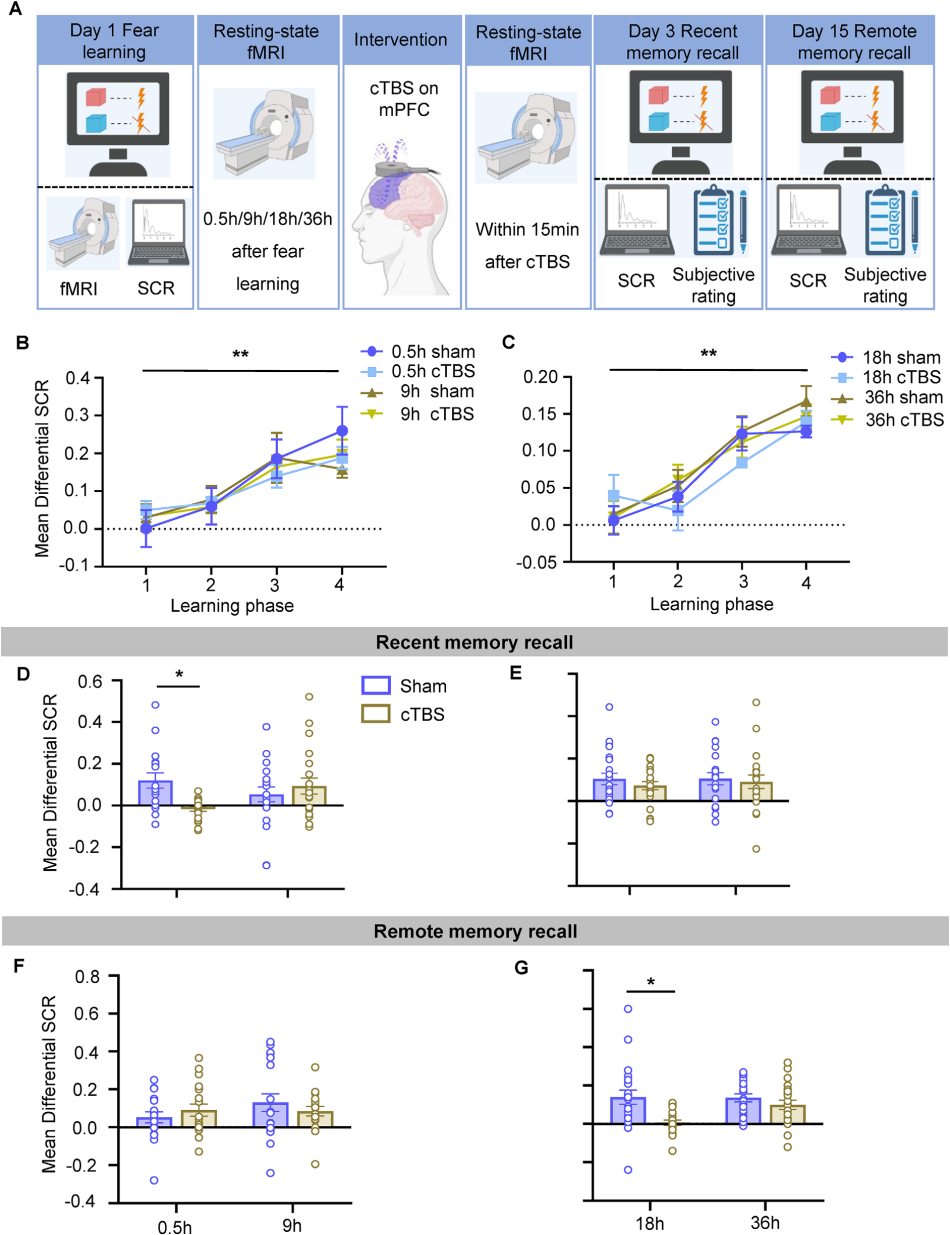
cTBS over mPFC during the delayed activation phase attenuates remote fear memory expression. (A) Timeline. (B) Fear learning in 0.5 and 9 h groups. Participants successfully acquired fear memory to CS+, with no group differences. Main effects of group (time window 0.5 h vs. 9 h: *F*_(1, 17)_ = 0.05, *p* = 0.83) and intervention type (cTBS vs. Sham: *F*_(1, 17)_ = 0.17, *p* = 0.68) were not significant. A significant main effect of learning session was detected (*F*_(3, 15)_ = 29.81, *p* < 0.01), with no significant interaction (*F*_(3, 15)_ = 1.04, *p* = 0.41). Post hoc analysis revealed that SCR was significantly higher in the last learning session than in the first and second sessions (Bonferroni, *p* < 0.01). (C) Fear learning in the 18 and 36 h groups. Participants successfully acquired fear memory toward CS+, with no group differences. Main effects of group (time window: 18 h vs. 36 h: *F*_(1, 19)_ = 3.62, *p* = 0.07) and intervention type (cTBS vs. Sham: *F*_(1, 19)_ = 0.34, *p* = 0.57) were not significant. A significant main effect of learning session was observed *F*_(3, 17)_ = 23.88, *p* < 0.01), with no significant interaction (*F*_(3, 17)_ = 1.53, *p* = 0.24). Post hoc tests showed that SCR was significantly higher in the last session than in the first session (Bonferroni, *p* < 0.01). (D) Recent fear memory recall test in the 0.5 and 9 h groups. A significant main effect was found (*F*_(3, 73)_ = 6.58, *p* = 0.02). Post hoc tests revealed significantly reduced SCR in the 0.5 h cTBS group compared with the 0.5 h sham group (Bonferroni, *p =* 0.02; one outlier was removed in the 0.5 h sham group based on the IQR boxplot method). (E) Recent fear memory recall test in 18 and 36 h groups. No significant difference was found (*F*_(3, 81)_ = 0.33, *p* = 0.80). Participants showed no difference in the recent memory recall test after the 18‐ or 36 h intervention. (F) Remote fear memory recall test in 0.5 and 9 h groups. No significant main effect was detected (*F*_(3, 69)_ = 0.87, *p* = 0.46). (G) Remote fear memory recall in the 18 and 36 h groups. A significant main effect was found (*F*_(3, 79)_ = 4.16, *p* < 0.01). Post hoc tests revealed significantly reduced SCR in the 18 h cTBS group compared with the 18 h sham group (Bonferroni, *p* < 0.01). *n* = 18–22 per group. Data are expressed as mean ± SEM. ^*^ *p* < 0.05, ^**^ *p* < 0.01.

Results showed that all participants successfully learned fear memory, as evidenced by the skin conductance response (SCR) and subjective ratings (Figure 5B,C and Figure S17A, B). No significant differences in demographic input were observed among the groups (Tables S1–S3). Participants who received cTBS at 0.5 h post‐learning showed significantly reduced fear responses during recent but not remote recall compared with the sham group (Figure 5D,F and Figures S18A and S19A). In contrast, participants who received cTBS at the 18 h timepoint demonstrated significantly reduced fear responses during remote but not recent recall compared with the sham group (Figure 5E,G and Figures S18C and S19C). Furthermore, all the participants except for those in the 18 h cTBS group showed significantly higher fear responses in the first phase of remote recall tests compared with their fear responses in the last phase of recent recall tests (Figure S19). No significant differences were detected between the 9‐ and 36 h cTBS groups and their respective sham groups (Figures S18B, D and S19B, D).

These results demonstrate that mPFC interference during the early phase specifically impaired recent fear memory, whereas interference during the delayed phase specifically weakened remote fear memory. This temporal dissociation is consistent with our animal results and supports time‐dependent roles of mPFC engagement in the systems consolidation of fear memory processing.

### 2.6 Enhanced mPFC Activity and Functional Connectivity Occur During the Delayed Consolidation Phase of the mPFC in Humans

To clarify how the mPFC supports memory retention, we analyzed fMRI data acquired during fear learning and at the early (0.5 h) and delayed (18 h) post‐learning windows. We found that mPFC activity significantly increased when presenting CS+ compared with CS‐ during fear learning across all participants (Figure 6A, demographic information in Table S1). During both the early and delayed phases, the mPFC exhibited significantly higher neural activity (amplitude of low‐frequency fluctuations, ALFF) compared with the 9‐ and 36 h control groups (Figure 6B,C,E,F and Table S6). Notably, the early phase saw enhanced spontaneous neural activity in the vmPFC. In the delayed phase, however, spontaneous activity was upregulated in the dmPFC, while vmPFC activity showed only a non‐significant trend toward elevation. To further understand the functional connectivity (FC) between the vmPFC and the dmPFC, we used the dmPFC as a seed region and observed that FC between the dmPFC and vmPFC was significantly enhanced during fear learning (Figure 6D, Figure S20A and Table S6). Additionally, FC between dmPFC and vmPFC was enhanced compared with control groups during both early and delayed phases (Figure S20B, C).

**FIGURE 6:**
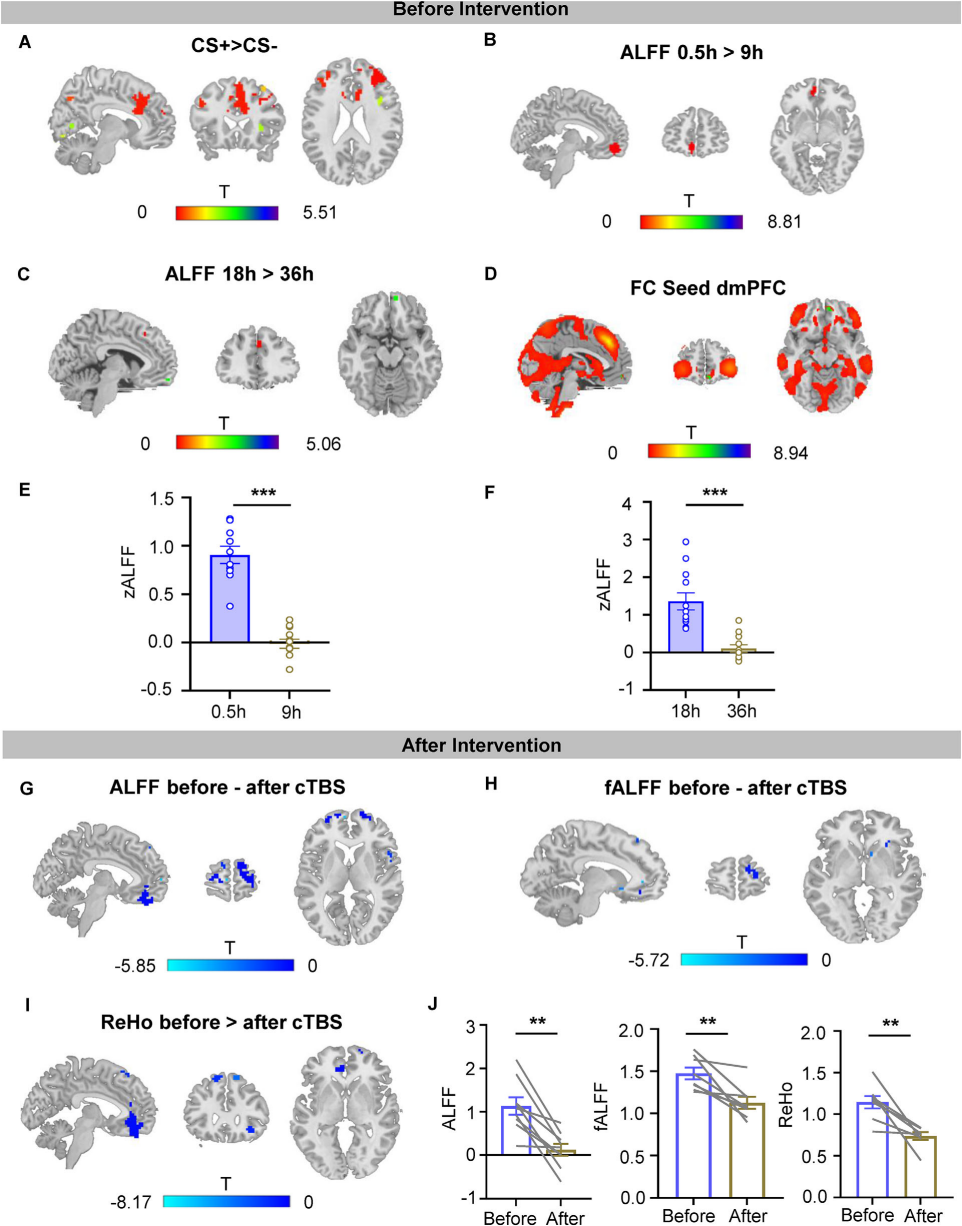
mPFC showed enhanced activity and functional connectivity during the delayed time window in humans. (A) BOLD responses during fear learning. The mPFC exhibited significantly higher activation to CS+ compared with CS‐ (*t*_(86)_ = 5.51, *p <* 0.01, FDR correction). (B, C) Spontaneous neural activity in the 0.5, 9, 18, and 36 h groups. The mPFC exhibited significantly higher amplitude of low‐frequency fluctuations (ALFF) in the 0.5 h group compared with the 9 h group (*t*_(33)_ = 8.81, *p* = 0.02, FDR correction), and significantly higher ALFF in the 18 h group compared with the 36 h group (*t*_(50)_ = 5.06, *p* = 0.03, FDR correction). (D) Functional connectivity (FC) during fear learning. Functional connectivity between the dmPFC and vmPFC was higher during fear learning (*F*_(2, 172)_ = 7.19, *p <* 0.01, FDR correction). (E) Statistical analysis of spontaneous neural activity in the 0.5 and 9 h groups. The vmPFC showed higher ALFF activity in the 0.5 h group compared with the 9 h group. No significant difference in dmPFC activity was observed between the two groups, and dmPFC activity was relatively weak. (F) Statistical analysis of spontaneous neural activity in the 18 and 36 h groups. The dmPFC showed higher ALFF activity in the 18 h group than in the 36 h group, whereas the vmPFC did not differ significantly. (G–J) Spontaneous neural activity after intervention. The mPFC exhibited significantly weaker neural activity following intervention. (G) ALFF; (H) fractional amplitude of low‐frequency fluctuations (fALFF); (I) regional homogeneity (ReHo). (J) Statistical analysis of spontaneous neural activity before and after intervention (left: ALFF, *t*_(14)_ = −5.85, *p <* 0.01, FDR correction; middle: fALFF, *t*_(14)_ = −4.92, *p* = 0.04, FDR correction; right: ReHo, *t*_(14)_ = −6.28, *p <* 0.01, FDR correction). Data are expressed as mean ± SEM. ^*^ *p* < 0.05, ^**^ *p* < 0.01, ^***^ *p* < 0.001.

Finally, to validate the efficacy of cTBS intervention, we conducted a comparative analysis of neural responses pre‐ and post‐intervention. The mPFC demonstrated significantly attenuated neural activity following the intervention, as indicated by decreased ALFF, fractional amplitude of low‐frequency fluctuation (fALFF), and regional homogeneity (ReHo) (Figure 6G–J and Table S6). Collectively, these findings indicate that the human mPFC not only exhibits enhanced local activity but also reinforces internal connectivity during the delayed phase, suggesting a conserved temporal pattern for mPFC in remote memory.

## 3 Discussion

Remote memory formation requires a complex interplay across multiple cortical and subcortical regions, with the mPFC playing a critical role [12, 13]. However, the temporal profile of prefrontal engagement in supporting remote memory remains unclear. In the present translational study from rats to humans, we identified a previously unrecognized delayed consolidation time window in the mPFC following fear conditioning that is essential for remote memory expression. This delayed phase, which occurs approximately 18 h after training, relies on mTOR‐dependent protein synthesis and functional projections from the VTA to the mPFC. By contrast, the early consolidation phase depends on β‐adrenergic signaling and is critical for the retrieval of recent memory. Notably, neuronal ensembles formed during the delayed phase are largely different from those engaged during the early phase, and are critical to the expression of remote memory. Similarly, non‐invasive stimulation of homologous mPFC regions in humans during the delayed consolidation phase selectively reduced remote fear memory, highlighting an evolutionarily conserved time window for persistence of remote memory (Figure 7).

**FIGURE 7:**
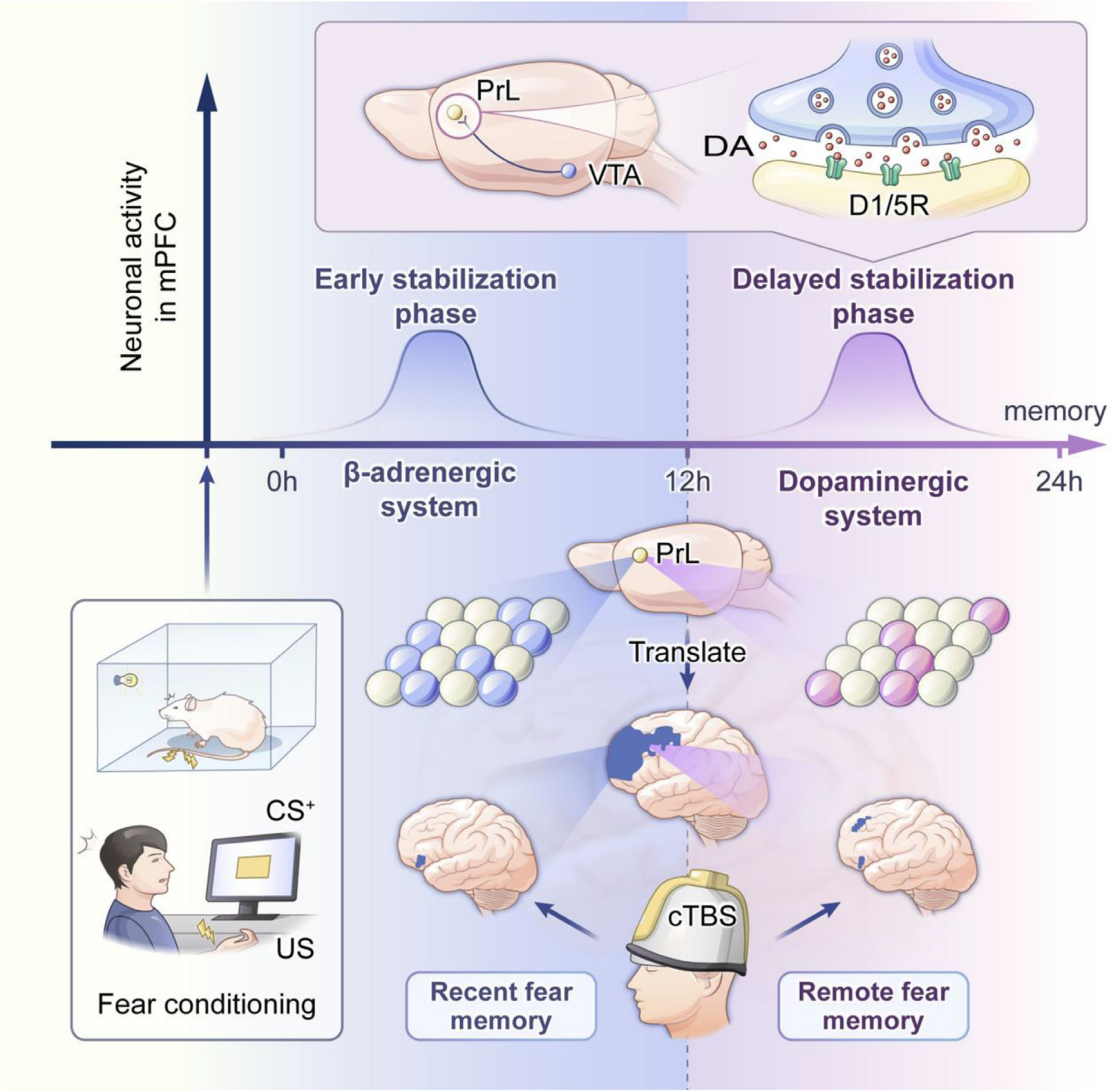
A temporal dissociation working model of mPFC function following fear memory formation. Immediately following fear memory acquisition, mPFC activity exhibits early and delayed phases of enhancement. The early phase occurs approximately 0.5–1.5 h post‐training, relies on β‐adrenergic signaling, and contributes specifically to recent memory recall. In contrast, the delayed phase takes place around 18 h post‐training, requires dopaminergic projections from the VTA to the mPFC, and is crucial for remote memory formation. Notably, different neuronal ensemble populations are recruited during these two phases. This temporal dissociation model of mPFC function is evolutionarily conserved in humans, as non‐invasive stimulation of the human homologue of the mPFC during these phases can modulate the expression of recent and remote memory, respectively.

Remote memories are widely considered to be primarily stored in cortical regions such as the mPFC through gradual information transfer from subcortical regions [6]. However, the role of the mPFC in organizing recent and remote memories appears more complex than previously thought. Our results are consistent with previous studies demonstrating that early cortical activity following fear memory encoding is critical for remote fear memory storage and maintenance [12, 13]. In addition, evidence also indicates that distinct inputs and outputs of the prelimbic cortex differentially contribute to recent vs. remote memory recall [27]. A recent study further reported that engrams in the mPFC recruited during contextual fear conditioning mediate both recent and remote memory recall, with distinct superficial and deep‐layer sub‐engrams differentially activated during memory retrieval [14]. This is highly consistent with our present observation that early c‐Fos expression is distributed across PrL layers, whereas delayed c‐Fos expression is concentrated in layers 5b–6 (Figure 1C and Figure S1). Together, these studies support a model in which PrL engrams contribute to both recent and remote memories through layer‐specific recruitment and input‐output‐specific circuit modulation, in agreement with our finding that early‐ and delayed‐phase ensembles are largely non‐overlapping.

Our findings indicate that delayed activation of neuronal ensembles after fear conditioning mediates the expression of remote fear memory, a result that diverges from earlier work in mice demonstrating that training‐encoded engram cells within the mPFC gradually mature over time and become reactivated during remote memory recall [13]. However, this discrepancy could stem from variations in the labeling time window. In addition, a recent study showed that, during systems consolidation, originally encoded mPFC neurons actively recruit additional, previously non‐activated neurons into the engram network through activity‐dependent synaptic strengthening [28]. These newly recruited neurons form functional monosynaptic connections with the original neuronal population and are reactivated during remote memory recall, indicating that engram maturation involves not only the strengthening of existing neurons but also the hierarchical recruitment of new neuronal assemblies guided by the original engram. Within this framework, the largely non‐overlapping nature of delayed‐activated PrL neuronal ensembles we observed could be accounted for by a model in which early‐activated PrL neuronal ensembles function as a foundational template governing the subsequent recruitment of their delayed‐activated counterparts. Nevertheless, the specific mechanisms by which the delayed‐activated neuronal ensembles are recruited to mediate the expression of remote fear memories remain to be investigated.

Notably, the functional involvement of mPFC activity during the early and delayed temporal windows also differs in a task‐specific manner. Our results indicated that early inhibition of protein synthesis in the PrL selectively disrupted recent memory in CFC, a hippocampus‐dependent task, but had no effect on recent memory in AFC (auditory fear conditioning), a primarily amygdala‐dependent, hippocampus‐independent task [29] (Figure 1I, K and Figure S8). This double dissociation indicates that the early mPFC activity is required for the consolidation of recent hippocampus‐dependent contextual fear memory, but not for recent auditory fear memory. In contrast, the delayed activity of mPFC appears critical for remote memory storage across both contextual and auditory fear memories, suggesting a more general role of the late mPFC time window in systems consolidation of remote fear memory regardless of initial hippocampal dependence [6]. This task‐specific recruitment of early vs. delayed mPFC mechanisms further highlights the different functional roles of these two temporal windows in recent vs. remote fear memory processing.

Neuromodulatory systems influence diverse behaviors, including reward processing, fear responses, learning, and memory formation [30, 31, 32]. Norepinephrine is required for both consolidation and reconsolidation of fear memory [20, 33], while dopamine contributes to the formation of long‐term fear memory [7, 34]. The distinct characteristics observed between early and delayed consolidation phases of mPFC activity—specifically, the early phase contributing to recent fear memory expression and the delayed phase regulating remote fear memory expression—suggest that these phases may be differentially regulated by specific neuromodulatory mechanisms. Our findings support this hypothesis, indicating that the early and delayed phases function through noradrenergic and dopaminergic signaling, respectively. This differential regulation provides important insights into the neurochemical basis of memory processing over time. However, the potential involvement of additional neuromodulatory systems in these phases requires further investigation.

We also found that the D_1_/D_5_ receptor‐dependent VTA‐PrL projection during the delayed consolidation phase is essential for the persistence of remote memory. One possibility is that delayed‐phase dopamine is released from the VTA onto PrL D_1_/D_5_ receptors. This finding aligns with previous research demonstrating dopamine's role in the VTA for maintaining fear memory [7] and addiction‐related memory [35] during late consolidation in the hippocampus. Alternatively, this VTA‐PrL projection might converge with other neural pathways during delayed PrL activation. These pathways may include projections from the lateral habenula and brainstem median raphe region, which are involved in processing aversion‐related and negative experiences [36, 37]. Additional convergence may occur with inputs from the hippocampus and basolateral amygdala (BLA), which are necessary for engram maturation in the PrL [12, 13]. In this scenario, during the delayed phase of the PrL, the VTA‐PrL input could integrate these multiple projections by recruiting specific PrL neurons into a coherent memory trace. More precisely, projections from the VTA to the mPFC and D_1_/D_5_ receptors in the mPFC may function by temporally synchronizing neural oscillations between the mPFC and amygdala, thereby facilitating aversive learning and behavior [38].

Conventionally, transcranial magnetic stimulation (TMS) has been applied to superficial brain regions (e.g., the dorsolateral prefrontal cortex) to modulate fear memory in humans [23, 39, 40], leaving deeper tissues unaffected. In the present study, we applied a cTBS pattern delivered through an H7 deep‐TMS coil [41, 42] to stimulate the mPFC, providing a more efficient approach for modulating deeper brain structures. We also investigated the effectiveness of this intervention by comparing neural responses before and after cTBS treatment and observed significantly reduced mPFC neural activity. When analyzing neural activity changes in mPFC subregions during memory processing, we observed increased vmPFC activity during the early phase and significantly enhanced functional connectivity between vmPFC and dmPFC during both early and delayed phases. Previous research has found that the primary brain regions storing memory traces vary over time, with the vmPFC mainly affecting long‐term memory preservation [43]. Additionally, studies have provided compelling evidence that theta oscillations within and between the vmPFC, dmPFC, and amygdala constitute a general mechanism of fear learning [44], which is consistent with our results regarding the role of the mPFC in fear memory processing. However, further research is needed to elucidate the neural network profiles underlying early and delayed consolidation phases.

Collectively, we identified a discrete late consolidation phase in the mPFC that is critical for the storage of remote fear memory. Targeting this window may obviate the need for intervention during the acute post‐trauma period and avoid re‐exposure to distressing cues inherent in treatments such as exposure therapy [45, 46]. By leveraging delayed mPFC plasticity with non‐invasive stimulation, we propose promising therapeutic possibilities for treating fear and trauma‐related disorders that minimize the risk of secondary trauma.

## 4 Materials and Methods

### 4.1 Animal Study

Adult male Sprague‐Dawley rats (220–240 g) were purchased from Beijing Vital River Laboratory Animal Technology Co., Ltd. Upon arrival, rats were housed in groups of four per cage and later individually housed post‐surgery under controlled conditions of temperature (22°C ± 2°C) and humidity (50% ± 5%), with a 12 h/12 h light/dark cycle (lights off at 8:00) and ad libitum access to food and water. All behavioral experiments, including fear conditioning training, were performed during the animal's dark period (8:00–20:00) to align with their active period, with consistent timing relative to the circadian cycle to avoid confounding effects of circadian rhythms. Specifically, fear conditioning training for the 18 h group was conducted during the dark phase (14:00–20:00), and subsequent c‐Fos immunostaining or interventions were performed the next morning (8:00–14:00). For the 12 h control group, fear conditioning was restricted to the late dark phase (19:30–20:00), with immunostaining performed at 8:00 the following day (light phase). We performed the experimental procedures in accordance with the National Institutes of Health Guide for the Care and Use of Laboratory Animals, and the procedures were approved by the Biomedical Ethics Committee for Animal Care and Use and Protection of Peking University (No. DLASBF0109).

### 4.2 Catheter Implantation

All of the rats were acclimated to the housing environment for 7 days before surgery. Surgical anesthesia was induced with 4% isoflurane in an air/oxygen mixture. The rats were intubated and secured in a stereotaxic frame. The rats were maintained at a surgical level of anesthesia with 1.5% isoflurane and carrier gases during surgery. Four jeweler screws were implanted in the skull using a Kopf stereotaxic apparatus, and then two stainless‐steel cannulas were inserted bilaterally in the PrL (anterior/posterior, + 2.9 mm; medial/lateral, ± 2.3 mm; dorsal/ventral, – 3.0 mm; 16° angles of inclination). Dental cement was applied to stabilize the implants. Obturators were inserted in the guide cannulas to prevent clogging. The rats were then allowed 7 days to recover from surgery and handled daily during the recovery period.

### 4.3 Stereotaxic Surgeries

The rats were deeply anesthetized with isoflurane. They were then placed in a stereotaxic apparatus. The virus solution was infused with a glass pipette. The volume of virus per injection site was 400 or 500 nL. After the injection, the pipette was left in the injection site for an additional 10 min to ensure viral particle diffusion.

To label the VTA‐PrL circuit, Retro‐AAV‐EF1α‐EGFP‐PA (500 nL) was bilaterally infused into the PrL (anterior/posterior, + 3.2 mm; medial/lateral, ± 0.5 mm; dorsal/ventral, – 4.0 mm). For chemogenetic regulation of the VTA‐PrL projection, AAV‐EF1α‐hM3Dq‐mCherry‐WPRE (400 nL), AAV‐EF1α‐hM4Di‐mCherry‐WPRE (400 nL), and AAV‐EF1α‐mCherry‐WPRE‐PA (400 nL) were bilaterally infused in the VTA (anterior/posterior, ‐4.8 mm; medial/lateral, ± 0.8 mm; dorsal/ventral, – 8.5 mm), and Retro‐AAV‐hSyn‐EGFP‐Cre‐WPRE‐hGH PA (500 nL) was bilaterally infused into the PrL (anterior/posterior, + 3.2 mm; medial/lateral, ± 0.5 mm; dorsal/ventral, – 4.0 mm). The accuracy of VTA‐PrL labeling was validated.

All viruses were provided by BrainVTA (Wuhan, China) or Braincase (Wuhan, China). Viral titers were 5.00 × 10^12^ vg/mL for Retro‐AAV‐EF1α‐EGFP‐PA, 5.00 × 10^12^ vg/mL for Retro‐AAV‐hSyn‐EGFP‐Cre‐WPRE‐hGH PA, 2.00 × 10^12^ vg/mL for AAV‐EF1α‐hM3Dq‐mCherry‐WPRE, AAV‐EF1α‐hM4Di‐mCherry‐WPRE, and AAV‐EF1α‐mCherry‐WPRE‐PA. Viruses were stored at −80°C, and rats were allowed a 21‐day recovery period in their home cages for viral expression, with daily handling.

### 4.4 Activity‐Dependent Neuronal Labeling

To enable labeling of neurons activated during the early phase of contextual fear conditioning (Figure 2A–C), we utilized two distinct genetic labeling paradigms. Initially, we stereotaxically injected a viral mixture containing AAV9‐c‐Fos‐tTA (500 nL) and AAV9‐TRE‐EGFP (500 nL) into the mPFC/PrL of the rats. Animals were maintained on doxycycline (Dox)‐containing food pellets (3000 mg/kg) throughout the experiment. Doxycycline was withdrawn 47.5 h prior to contextual fear conditioning by replacing Dox‐containing food with regular food pellets. Doxycycline administration was resumed 0.5 h post‐conditioning by returning animals to Dox‐containing food and administering an intraperitoneal (i.p.) injection of Dox (50–100 mg/kg).

To independently label mPFC neurons during the same early phase using an alternative genetic strategy (Figure S9), we bilaterally injected a viral mixture containing AAV9‐c‐Fos‐ERT2CreERT2 (500 nL) and AAV9‐hSyn‐DIO‐mCherry (500 nL) into the PrL of rats. Following a three‐week viral expression period, 4‐OHT was administered intraperitoneally 0.5 h post‐conditioning.

Neurons labeled by these two independent systems were subsequently subjected to co‐labeling analysis with endogenous c‐Fos expression at 18 h post‐conditioning, thereby enabling the precise temporal mapping of neuronal activation profiles within the mPFC/PrL circuitry.

To fluorescently label neurons activated at 18 h, we bilaterally injected a viral mixture containing AAV9‐c‐Fos‐ERT2CreERT2 (500 nL) and AAV9‐hSyn‐DIO‐mCherry (500 nL) into the PrL of SD rats. Following a three‐week viral expression period, we administered 4‐OHT intraperitoneally at 18 h post‐conditioning. Memory tests were conducted 2 or 14 days post‐initial training.

For selective manipulation of neurons activated 18 h after training, a viral mixture of AAV9‐c‐Fos‐ERT2CreERT2 (500 nL) and AAV9‐hSyn‐DIO‐hM4Di /hM3Dq‐mCherry (500 nL) was bilaterally injected into the PrL of SD rats. Following a three‐week viral expression period, 4‐OHT was administered intraperitoneally at 18 h post‐conditioning. To inhibit/activate the 18h‐labeled neurons during memory retrieval, CNO was administered intraperitoneally 30 min prior to testing on day 2 or day 14.

For optogenetic activation of neurons activated 18 h after training, a viral mixture of AAV9‐c‐Fos‐ERT2CreERT2 (500 nL) and AAV9‐hSyn‐DIO‐hChR2(H134R)‐mCherry (500 nL) was bilaterally injected into the PrL of SD rats.

All viruses, provided by Shandong Vigene Biosciences Inc (Shandong, China) or BrainVTA (Wuhan, China), had titers of 2.16 × 10^12^ vg/mL for AAV9‐c‐Fos‐tTA, 3.14 × 10^12^ vg/mL for AAV9‐TRE‐EGFP, 2.45 × 10^12^ vg/mL for AAV9‐c‐Fos‐ERT2CreERT2, 4.71 × 10^12^ vg/mL for AAV9‐hSyn‐DIO‐mCherry, 4.27 × 10^12^ vg/mL for AAV9‐hSyn‐DIO‐hM4Di‐mCherry, 5.05 × 10^12^ vg/mL for AAV9‐hSyn‐DIO‐hM3Dq‐mCherry, and 5.02 × 10^12^ vg/mL for AAV9‐hSyn‐DIO‐ hChR2(H134R)‐mCherry. All viruses were stored at −80°C.

### 4.5 Drugs and Drug Delivery

Anisomycin (62.5 µg/µL; Sigma–Aldrich) was dissolved in equimolar HCl and sterile saline and adjusted to pH 7.4 with NaOH (1.2 µL/side). Rapamycin (1 µg/µL; LC Laboratories) was dissolved in 80% dimethylsulfoxide and 20% sterile saline (0.7 µL/side). Propranolol (30 µg/µL; Sigma) was dissolved in sterile saline (1.2 µL/side). SCH23390 (1 µg/µL; Tocris) was dissolved in sterile saline (0.7 µL/side). SKF38393 (25 µg/µL; Sigma) was dissolved in sterile saline (0.7 µL/side). Drugs and vehicles were microinjected bilaterally into the PrL at a rate of 0.25 µL/min using Hamilton syringes that were connected to 30‐gauge injectors (Plastics One, Roanoke, VA, USA) extending 1 mm below the guide cannula. The needle was kept in place for an additional 1 min to allow diffusion.

Clozapine N‐oxide (CNO, MedChemExpress) was dissolved in 5% DMSO and 95% sterile saline (1.5–2 mg/kg, i.p.). 4‐hydroxytamoxifen (4‐OHT, MedChemExpress) was dissolved in minimal ethanol and suspended in corn oil at a concentration of 50 mg/mL, at which time the ethanol was completely removed (50–75 mg/kg, i.p.). Doxycycline hyclate (Dox, MedChemExpress) was dissolved in sterile saline (50–100 mg/kg, i.p.).

### 4.6 Contextual Fear Conditioning and Freezing Test

Fear conditioning experiments were conducted using the Startle and Fear‐conditioning System (Panlab, Barcelona, Spain). The apparatus comprised a conditioning cage with a grid floor wired to a shock generator, surrounded by an acoustic chamber. The chambers were cleaned with 75 % alcohol, and the jasmine scent was sprayed into the cage between the conditioning of each rat to remove the odor left by the previous rat. On the training day, rats were placed individually in the chamber and allowed to explore it for 2 min, after which they received a 1 s foot shock (0.9 mA for strong training, 0.3 mA for weak training). Two additional identical foot shocks were given at an interval of 2 min between each shock. After the last foot shock, the rats were allowed to explore the chamber for an additional 1 min before being returned to their home cage. Freezing behavior, defined as the absence of all movement except respiration, was assessed in the same training chamber for 5 min without foot shocks, using weight transducers and an automated scoring system (Packwin 2.0 software). Calibration of load cells in each experimental box was performed initially and periodically to ensure consistent sensitivity across all experiments. The calibration process included zeroing the weight transducers for optimal performance and repeating calibration steps for each chamber.

### 4.7 Auditory Fear Conditioning and Freezing Test

Auditory fear conditioning was also conducted using the Startle and Fear‐conditioning System (Panlab, Barcelona, Spain). On the day preceding conditioning, rats were acclimated to training context A for 10 min. On the conditioning day, rats were habituated for 2 min in the chamber and then received three presentations of a 20 s auditory tone (80 dB, cage enclosed) co‐terminating with a 1 s, 0.9 mA foot shock, with a 120 s intertrial interval. Following the last foot shock, rats were allowed to explore the chamber for an additional 1 min before returning to their home cage. Freezing behavior was evaluated during silence and CS presentation.

The auditory fear memory test was performed in a different chamber (context B) for 360 s, starting with a 120 s habituation period followed by presentation of three CSs without foot shock (US), each separated by 90 s. Freezing responses to the CS and contextual background were quantified as percentages of total CS and habituation periods, respectively, using weight transducers and an automated scoring system (Packwin 2.0 software).

### 4.8 Optogenetic Activation of PrL Engram Cells

In this study, we tested whether activation of PrL engram cells labeled with ChR2–mCherry during the delayed activation phase after CFC was sufficient to drive memory recall at recent and remote time points. For light‐induced freezing behavior tests, a context distinct from the CFC training chamber was used. Chamber ceilings were customized to hold a rotary commutator connected to two 0.32 m patch cords. All rats had patch cords connected to their optical fiber implants before testing. ChR2 was stimulated at 4 Hz (15 ms pulse width) or 20 Hz (15 ms pulse width) using a 473 nm laser (10–15 mW) during designated epochs. Testing sessions were 12 min in duration, consisting of four 3 min epochs, with the first and third as light‐off epochs and the second and fourth as light‐on epochs. At the end of the 12 min session, each mouse was detached and returned to its home cage. Chamber floors were cleaned with 70% ethanol before each test. Experiments that produced significant behavioral effects were replicated three times in the laboratory. Following behavioral testing, brain sections were prepared to confirm adequate viral labeling in target areas. Animals with insufficient labeling were excluded prior to behavioral quantification.

### 4.9 Locomotor Activity

Locomotor activity was assessed by placing rats individually in the locomotor chamber (40 cm × 40 cm × 65 cm) for 10 min, with activity recorded and analyzed using an automated video tracking system (DigBehv‐LM4; Shanghai Jiliang Software Technology, Shanghai, China). Total distance traveled (in centimeters) during the test period was calculated.

### 4.10 Immunostaining

Brains were fixed with 4% paraformaldehyde and transferred through graded sucrose solutions (30%) in phosphate‐buffered saline (PBS; pH 7.2). Sections were washed with PBS, blocked with 5% BSA with 0.2% Triton X‐100 for 1 h at room temperature, and then incubated overnight at 4°C with primary antibodies, including anti‐c‐Fos (1:500, Cell Signaling Technology, catalog no. 2250s), anti‐CaMKII (1:500, Abcam, catalog no. ab22609), anti‐tyrosine hydroxylase (TH; 1:500, Millipore, catalog no. 3277994), and anti‐GAD67 (1:500, Abcam, catalog no. ab26116). After washing with PBS, sections were incubated with fluorescent‐conjugated secondary antibodies, including goat anti‐rabbit secondary antibody (Alexa Fluor 488, 1:500, Invitrogen, catalog no. A‐21206), donkey anti‐mouse secondary antibody (Alexa Fluor 488, 1:500, Invitrogen, catalog no. A32723), donkey anti‐mouse secondary antibody (Alexa Fluor 594, 1:500, Invitrogen, catalog no. A‐21203), and goat anti‐rabbit secondary antibody (Alexa Fluor 647, 1:500, Invitrogen, catalog no. A48285), which were dissolved in blocking buffer for 2 h at room temperature followed by washing with PBS. Finally, the sections were mounted with a medium containing DAPI (Vector Laboratories, catalog no. H2000) and stored at 4°C for further analysis. Fluorescence microscopy was performed using an Olympus microscope (Tokyo, Japan) equipped with a 20 × objective lens for image acquisition.

Cell counting of immunofluorescence data was conducted by an investigator blinded to experimental group assignments, utilizing ImageJ software (NIH, Bethesda, MD). For each rat, a minimum of three non‐adjacent sections per brain region were analyzed. Immunopositive cells were quantified using an automated thresholding protocol consistently applied across all sections within each brain region. Double‐labeled cells were identified through manual verification of co‐expression in cells pre‐selected by ImageJ's automated detection algorithm.

### 4.11 In Situ Hybridization

Brains were fixed in 4% paraformaldehyde and dehydrated through a graded series of sucrose solutions (final concentration 30%) in phosphate‐buffered saline (PBS; pH 7.2). RNAscope HiPlex assays were performed using the RNAscope HiPlex12 Kit (Advanced Cell Diagnostics, 324443) and HiPlexUp Reagent (ACD, 324190). For sectioning, brains (with cerebellum removed) were frozen in Tissue‐Tek OCT compound and sectioned at 15 µm with a cryostat. Sections were fixed in 4% neutral buffered formalin, dehydrated with graded ethanol, treated with protease, and incubated with c‐Fos target probes (Bio‐Techne, Lot: 26146A) in a HybEZ Oven for 2 h at 40°C. Signal amplification was performed via hybridization with HiPlex Amp 1–3 for 30 min at 40°C. Probe signals were labeled with fluorophores using Opal 570 (ACD, ASOP570) for 15 min at 40°C. Cell nuclei were counterstained with DAPI. Fluorescence images were acquired with an Olympus microscope (Tokyo, Japan) equipped with a 20 × objective lens. Data analysis methods for in situ hybridization were identical to those described for immunofluorescence.

### 4.12 Whole‐Cell Electrophysiology

For whole‐cell voltage‐clamp electrophysiological recordings, 300 µm horizontal PrL slices were prepared in an ice‐cold cutting solution that contained 87 mm NaCl, 3.0 mm KCl, 1.5 mm CaCl_2_, 1.3 mm MgCl_2_, 1.0 mm NaH_2_PO_4_, 26 mm NaHCO_3_, 20 mm D‐glucose, and 75 mm sucrose, and saturated with 95% O_2_ and 5% CO_2_. After incubation for 30 min at 32°C, the slices were recovered at room temperature for > 30 min in ACSF solution (124 mm NaCl, 3.0 mm KCl, 2 mm CaCl_2_, 1.3 mm MgCl_2_, 1.0 mm NaH_2_PO_4_, 26 mm NaHCO_3_, and 20 mm D‐glucose). The slices were transferred to a recording chamber and maintained at 30 °C–32°C and a flow rate of 2 mL/min, and PrL pyramidal neurons were held at −70 mV and recorded using a pipette solution that contained the following: 115 mm Cs methylsulfate, 5 mm CsCl, 4 mm ATP‐Mg, 0.5 mm GTP‐Na, 0.5 mm EGTA, 10 mm HEPES, and 4.0 mm QX‐314 [pH 7.2, 270–280 mOsm with sucrose]. For sEPSC recordings, the recording electrodes were about 2 MΩ. The series resistance, usually between 8 and 14 MΩ, was uncompensated. The series resistance was monitored regularly during the experiments. Data were discarded if series resistance exceeded 16 MΩ or the fluctuation of series resistance exceeded 15% during the recordings. Whole‐cell recordings were performed using a Multiclamp 700B amplifier (Molecular Devices, San Jose, CA, USA) and Axon Digidata 1440A (Molecular Devices, San Jose, CA, USA) controlled by pClamp 10 software (Molecular Devices, San Jose, CA, USA). The data were filtered at 4 kHz and digitized at 20 kHz. The data were analyzed offline using Axograph X.

For ex vivo pharmacology, drugs were bath applied: β‐adrenoceptor antagonist propranolol ((S)‐(−)‐propranolol hydrochloride, 295.80 g/mol; Sigma–Aldrich) at a concentration of 100 µm, D_1_/D_5_ receptor antagonist SCH23390 (SCH23390 hydrochloride, 324.24 g/mol; Tocris) at a concentration of 10 µm, D_1_/D_5_ receptor agonist SKF38393 ((R)‐(+)‐SKF38393 hydrochloride, 291.77 g/mol; Sigma) at a concentration of 20 µm, these drugs were freshly dissolved in ACSF. After a 2 min baseline recording, the drug‐containing ACSF was perfused in and waited for 10 min before recording sEPSC responses. The effect of the drug was determined by comparing the sEPSC of the same cell before and after the drug perfusion.

### 4.13 Western Blot Assay

Samples for the Western blot assay were prepared as previously described [7, 47]. Membranes were first incubated with anti‐c‐Fos antibody (1:1000; Abcam), then stripped and re‐probed with anti‐Actin antibody (1:5000; ZSGB‐BIO, China). Densitometry analysis was performed using ImageJ.

### 4.14 Human Study Design

We conducted a between‐subjects single‐blind experiment, with participants unaware of whether they received cTBS or sham intervention. Baseline demographic data were confirmed to be homogeneous across groups prior to the experiment. Eligible participants were randomly assigned to different groups based on the order in which they enrolled in the study. In addition, the fear learning task was conducted between 8:00 and 20:30, with each participant's specific experiment time arranged according to their assigned group. In both the 18 h cTBS group and 18 h sham group, participants typically underwent the fear learning task between 14:00 and 20:30, followed by TMS 18 h later, specifically between 8:00 and 15:00 the following day. In our experimental design, we considered the potential influence of different time windows on memory performance, and therefore endeavored to distribute test sessions as evenly as possible throughout the day.

In our experiments, participants underwent a fear conditioning procedure during fMRI scanning. The protocol for the fear learning and fear memory tests was based on previous studies [48, 49]. Colored squares (conditioned stimuli [CSs]) were presented during fMRI scanning. The electrical stimulation threshold for each participant was first determined by progressively increasing the electric shock intensity. Conditioned stimuli paired with electric shock (unconditioned stimulus [US]) were designated as CS+, whereas CSs not paired with shock were designated as CS–. Each CS was presented for 4 s with an inter‐trial interval of 10 s. The US co‐terminated with the CS and lasted 2 s, with a reinforcement rate of 33.3%. The fear acquisition procedure comprised 60 trials, with a total scanning duration of approximately 15 min. Participants underwent structural and resting‐state fMRI at 0.5, 9, 18, or 36 h after fear acquisition. A total of 87 participants were included in this phase (*n* = 23 in the 0.5 h group; *n* = 12 in the 9 h group; *n* = 31 in the 18 h group; *n* = 21 in the 36 h group). Further procedural details are provided in the Supporting Information. Demographic information is presented in Table S1.

Following fear acquisition, participants underwent resting‐state fMRI scanning and subsequently received TMS intervention, followed by recent and remote memory recall tests. In this phase, a total of 71 participants underwent fMRI scanning and 163 participants received TMS intervention (*n* = 20 in the 0.5 h cTBS group; *n* = 18 in the 0.5 h sham group; *n* = 20 in the 9 h cTBS group; *n* = 20 in the 9 h sham group; *n* = 22 in the 18 h cTBS group; *n* = 22 in the 18 h sham group; *n* = 21 in the 36 h cTBS group; *n* = 20 in the 36 h sham group). Within each time‐point group (0.5, 9, 18, and 36 h), participants were randomly assigned to receive either cTBS or sham intervention. Following intervention, a subset of participants underwent resting‐state fMRI scanning (*n* = 15). 58 h later and 14 days after fear acquisition, participants underwent the recent and remote recall tests, respectively. The recent recall test was a spontaneous recovery test (SR test), and the remote recall tests were the SR test and a reinstatement test. No US was delivered during the SR test. In the SR test, a total of 30 CSs were randomly presented; each CS lasted 4 s, with an inter‐CS interval of 8–12 s. In the reinstatement test, participants received four unsignaled electric shocks delivered at the onset, and the intensity of stimulation was slightly lower than the threshold for each participant. After the recall tests, participants provided subjective fear ratings for the CS+ and CS− by allocating 100 points between the two stimuli. We used ANOVA to compare subjective rating ratios (lgRating Score / lgRating Score Max) among groups to assess differential fear levels and quantify subjective fear responses (further details are provided in the Supporting Information). During the remote recall test (14 days post‐acquisition), some participants were lost to follow‐up (*n* = 2 in the 0.5 h cTBS group; *n* = 2 in the 9 h cTBS group; *n* = 1 in the 9 h sham group; *n* = 1 in the 18 h sham group; *n* = 1 in the 36 h sham group).

### 4.15 Ethics

The human study protocol was approved by the Peking University Biomedical Ethics Committee and West China Hospital of Sichuan University Biomedical Ethics Committee (Ethics Approval Number: 2020122817013952, 20221502). Written informed consent was obtained from all participants prior to enrollment.

### 4.16 Participants

We designed a single‐blind, between‐subjects experiment to investigate the role of late consolidation in recent and remote memory in healthy participants. They were recruited from Peking University and Sichuan University via online advertisements. Detailed inclusion and exclusion criteria are provided in the Supporting Information.

### 4.17 Transcranial Magnetic Stimulation Intervention

Active and sham (dTMS) were delivered using a TMS stimulator (Yingchi Co. Ltd.) equipped with an H7 coil (BrainsWay). The H‐coil utilizes the principle of vector superposition of induced electric fields and adopts a special helmet‐like structure to orient the stimulation target point tangentially to the scalp, thereby minimizing non‐tangent elements. This design enhances the induced electric field in deep target tissues while attenuating stimulation of non‐target tissues [41, 42, 50]. Upon coil discharge, the electric fields generated by coil windings at different positions spatially summate in deeper cortical layers, producing a broader and deeper induced electric field that enables effective deep brain stimulation [51]. In each session, the optimal spot on the scalp for stimulation of the leg motor cortex was localized. The resting motor threshold was defined as 80% of the active motor threshold. The coil was then moved 6 cm anterior to the motor spot for the mPFC intervention and tightened to the scalp. The continuous theta‐burst stimulation (cTBS) protocol consisted of 1800 pulses delivered in triplet bursts at a burst frequency of 50 Hz, repeated at 5 Hz. Sham stimulation was delivered using a figure‐of‐eight coil positioned over the vertex, localized according to the international standard EEG electrode 10/20 lead system [23, 52, 53, 54].

### 4.18 Psychophysiological Stimulation and Assessment

Electric shocks were delivered by a constant‐current STM200 stimulator (BIOPAC Systems, Goleta, CA, USA). Stimulation waveforms were programmed via E‐Prime software (Psychology Software Tools, Sharpsburg, PA, USA). Fear responses were assessed by SCR measured with the BIOPAC MP150 system and analyzed with AcqKnowledge 4.2.1 software (BIOPAC Systems, Goleta, CA, USA). Further details are provided in the Supporting Information.

### 4.19 Statistical Analysis

Demographic information, baseline questionnaire data, SCR data, and subjective ratings on the questionnaire were analyzed using SPSS software (SPSS, Chicago, IL, USA). Structural and resting‐state fMRI data were analyzed using MATLAB software (MathWorks, Natick, MA, USA). Further details are provided in the Supporting Information. Statistical analyses for the animal experiments were performed using SPSS software. Potential outliers were detected using the interquartile range (IQR) boxplot method. Data points outside 1.5 × IQR were defined as outliers. Group differences were initially assessed using one‐way or two‐way analysis of variance (ANOVA). Bonferroni‐corrected post hoc tests were used for post hoc comparisons across all groups. In addition, a priori planned comparisons using unpaired t‐tests or paired t‐tests were conducted between the control group and each experimental group based on our experimental hypotheses. All data are expressed as the mean ± standard error of the mean (SEM). Statistical details are presented in the figure legends. Values of *p* ≤ 0.05 were considered statistically significant.

## Author Contributions

**Lin Lu**, **Kai Yuan**, **Lu Cao**, **Yu‐Xin Zhang**: conceptualization. **Kai Yuan**, **Lu Cao**, **Yu‐Xin Zhang**, **Zi‐Hang Li**, **Tian‐Ye Jia**, **Xiang‐Dong Tang**, **Peng Li**, **Xiao Lin**, **Tang‐Sheng Lu**, **Wen Zhang**, **Shu‐Fei Feng**, **Ting‐Ting Li**: methodology. **Kai Yuan**, **Lu Cao**, **Yu‐Xin Zhang**, **Jia‐Hui Deng**: investigation. **Lu Cao**, **Yu‐Xin Zhang**, **Kai Yuan**, YH, **Tian‐Ye Jia**, **Jia‐Hui Deng**, **Xiao‐Xing Liu**: visualization. **Lin Lu**, **Kai Yuan**: Funding acquisition. **Lu Cao**, **Yu‐Xin Zhang**, **Kai Yuan**: project administration. **Kai Yuan**, **Lin Lu**, **Ying Han**: supervision. **Lu Cao**, **Yu‐Xin Zhang**, **Kai Yuan**: writing – original draft. **Kai Yuan**, **Ying Han**, **Yan‐Xue Xue**, **Jie Shi**, **Lin Lu**: writing – review & editing.

## Funding

This work was supported in part by the National Natural Science Foundation of China (no. 82288101, no. 82371499), the National Programs for Brain Science and Brain‐like Intelligence Technology of China (STI2030‐Major Projects, 2021ZD0200800), and the Young Elite Scientists Sponsorship Program by CAST (2023QNRC001).

## Conflicts of Interest

The authors declare no conflicts of interest.

## Supporting information




**Supporting File**: advs78000‐sup‐0001‐SuppMat.docx.

## Data Availability

All data needed to evaluate the conclusions in the paper are present in the paper and/or the Supporting Information.
