# Supplementary material for: A Critical Time Window of Neocortex Modulation for Remote Fear Memory in Rats and Humans
Source: Adv Sci (Weinh). 2026 Sep 30:e20707. Online ahead of print. doi: 10.1002/advs.202520707 (PMC13626168; doi:10.1002/advs.202520707)
Supplement: Supplementary file 1 [file ADVS-9999-e20707-s001.docx]

Supplementary Materials for

**A critical time window of neocortex modulation for remote fear memory in rats and humans**

Lu Cao *et al.*

*Corresponding author. Email: yuankai@pku.edu.cn; [linlu@bjmu.edu.cn](mailto:linlu@bjmu.edu.cn); yanxuexue@bjmu.edu.cn; [yinghan@bjmu.edu.cn](mailto:yinghan@bjmu.edu.cn); shijie@bjmu.edu.cn

**This file includes:**

**Supplementary materials and methods**

**Supplementary Figures and Figure Legends**

**Supplementary Table S1-S6**

**Supplementary Materials and Methods**

*Participants*

We designed a single-blind experiment to assess the effect of late consolidation on recent and remote memory in healthy participants. We recruited healthy participants at Peking University and Sichuan University through online advertising. Participants were randomly assigned to eight groups. All participants voluntarily signed an informed consent form. They were 18-35 years old, with more than 9 years of education. The exclusion criteria were participants who had mental disorders that met *Diagnostic and Statistical Manual of Mental Disorders*, 5th edition, diagnostic criteria; had severe physical diseases, such as confirmed diabetes, hypertension, heart disease, etc.; had previously taken psychotropic drugs, such as antidepressants or other drugs that are used to treat psychosis, and had not completed drug elution at the beginning of the study; exhibited excessive agitation and poor compliance; and had contraindications to transcranial magnetic stimulation (TMS).

*Imaging acquisition and preprocessing*

Imaging data were acquired using a 3.0 Tesla MR scanner (EXCITE HD GE Medical System, Milwaukee, WI, USA) with a 32-channel head coil. Data quality was supervised and strictly controlled by professional technicians throughout scanning. Functional images were obtained using a T2*-weighted echo-planar imaging (EPI) pulse sequence (Repetition time (TR) = 1200 ms, echo time (TE) = 29 ms, field of view (FOV) = 216 mm, flip angle (FA) = 60°, voxel size = 2.4 mm × 2.4 mm × 2.4 mm, slice number = 60, slice thickness = 2.4 mm). The total scanning time for resting-state functional images was 8 min. Structural T1 images were collected using three-dimensional spoiled gradient recalled images in a sagittal orientation (TR = 6700 ms, FOV = 256 mm × 256 mm, FA = 12°, voxel size = 1 mm × 1 mm × 1 mm). The total scanning time for structural T1 images was 10 min.

Functional images were preprocessed using Statistical Parametric Mapping 12 (University College London; http://www.fil.ion.ucl.ac.uk/spm) with slice timing, realignment, co-registration with T1, normalization into the Montreal Neurological Institute (MNI) template space, and smoothing using a Gaussian kernel of 6 mm full width at half maximum. We then removed linear drift, combined the six head motion parameters and cerebrospinal fluid and white matter signals with global signal regression, and filtered the data with a 0.01-0.08 Hz band pass filter to reduce the impact of low-frequency drift, head movements, and high-frequency respiratory and cardiac noise on the signals. After preprocessing, participants with excessive head movements (> 3 mm or 3°) and participants whose functional images could not be accurately registered into MNI space were excluded from the analysis.

*Psychophysiological stimulation and assessment*

Electric shocks were delivered by a constant-current STM200 stimulator (BIOPAC Systems, Goleta, CA, USA). The stimulating electrodes were connected to disposable electrocardiogram electrodes on the inner side of the left wrist. The disposable electrocardiogram electrodes were pasted to avoid the blood vessels of the participants. Electrical stimulation was nested in a program using E-Prime software (Psychology Software Tools, Sharpsburg, PA, USA). The trigger of electrical stimulation depended on the color of the squares (CSs) and reinforced intensity. The fear response was assessed by the SCR, which was recorded through shielded Ag-AgCl electrodes. The electrodes were connected to the pulp of the index and middle fingers of the left hand. Skin conductance response waveforms were measured using the BIOPAC MP150 system and analyzed using AcqKnowledge 4.2.1 software (BIOPAC Systems, Goleta, CA, USA).

*Behavioral statistical analysis*

*Demographic data analysis.* Demographic information (except for gender) and baseline questionnaire data were analyzed using analysis of variance (ANOVA) to compare baseline data among groups. For gender in each group, we conducted the *χ^2^* test. We performed *post hoc* analyses of significant effects in the ANOVAs using Bonferroni correction. Values of P *<* 0.05 were considered statistically significant.

*Skin conductance response data analysis.* For the SCR raw data, we recorded the peak-to-peak SCR change in the 2-10 s time window after each CS was shown. We standardized the SCR data through square-root transformation. We then could obtain the differential SCR by subtracting the data for the CS^–^ from the data for the CS^+^. The data in each group were then averaged, and then repeated-measures ANOVAs were conducted to compare differential SCRs among groups in each phase. For the analysis in the fear learning phase, the electric shock came after the CS^+^ with a reinforced intensity of 41.7%. These US trials evoked large, non-CS-related SCRs and were therefore excluded. The acquisition of fear memory was determined by the last three unreinforced CS^+^. An average differential SCR in the last three CS trials > 0.05 indicated successful fear memory learning. Furthermore, we divided fear learning into four sessions, and each session contained 5 pairs of unreinforced CS^+^ and CS^-^. We conducted repeated-measures ANOVAs to compare the SCR in all the four sessions to examine the effect of time. For the analysis of the recent and remote fear memory tests, the recall of fear memory was assessed by CS^+^ in the first three trials. In the recent and remote memory tests, the first stimulus (whether the CS^+^ or CS^–^) may cause panic or shock responses among the participants. Thus, in our analysis of the recent and remote memory tests, we excluded the data in the first trial. An average differential SCR in the first three CS trials (excluding the first CS) > 0.05 indicated that the participants still had strong fear responses to the CS^+^. Furthermore, we divided recent memory test into five sessions, and each session contained 3 pairs of CS^+^ and CS^-^. We conducted repeated-measures ANOVAs to compare the SCR in all the five sessions to examine the effect of time. We performed *post hoc* analyses of significant effects in the ANOVAs using Bonferroni correction.

*Subjective rating data analysis.* For the subjective rating questionnaire, we obtained scores for the CS^+^ and CS^–^ and the score ratio for each participant. We then conducted *t*-tests to compare rating scores for the CS^+^ and CS^–^. Finally, we conducted ANOVA to compare participants’ subjective rating ratios (lg_Rating Score_ / lg_Rating Score Max_) among groups and performed *post hoc* analyses of significant effects in the ANOVAs using Bonferroni correction.

*Imaging data analysis*

After excluding participants who did not meet the criteria, 87 participants remained for the fear learning phase and 71 participants for the different time windows before intervention.

During the fear learning phase, condition events were modeled using a canonical hemodynamic response function for reinforced conditioned stimuli (CS+) and unreinforced stimuli (CS–). To examine conditioning-induced activation, we compared neural responses between CS+ and CS– (i.e., CS+ > CS–). CS+ trials paired with electric shock were excluded because we focused on neural responses evoked by CS+ rather than somatosensory stimulation.

For the different time windows before intervention, we analyzed amplitude of low-frequency fluctuations (ALFF) to assess spontaneous neural activity. After band-pass filtering (0.01–0.08 Hz) and linear-trend removal, we used Fast Fourier Transform (FFT) to convert time series to the frequency domain and obtain the power spectrum. Because power at a given frequency is proportional to the square of the amplitude of that component, the power spectrum from FFT was square-rooted and averaged across 0.01–0.08 Hz for each voxel; this average was defined as ALFF. Spatial normalization, resampling (3 mm × 3 mm × 3 mm), and smoothing (full width at half maximum = 6 mm) were applied to ALFF maps. For normalization, ALFF of each voxel was divided by the global mean ALFF within a brain mask. The mask was derived from the intersection of all subjects’ T1 images. MRIcro software was used to remove non-brain tissue (Chris Rorden, http://www.psychology.nottingham.ac.uk/staff/cr1/mricro.html). The DPABI toolbox (https://rfmri.org/DPABI) was used for assisted processing.

We then performed region-of-interest (ROI) analysis for both the fear learning phase and pre-intervention time windows. We used the differential activation cluster from ALFF analysis as a seed region to compare CS+ > CS– responses during fear learning and spontaneous activation across time windows. A voxel-level threshold of P < 0.05, FDR-corrected was used to define significant activation.

After intervention, a subset of participants underwent resting-state fMRI scanning (total n = 15) to further assess changes in neural activity. In addition to ALFF, we analyzed fractional amplitude of low-frequency fluctuations (fALFF) and regional homogeneity (ReHo) using the DPABI toolbox. After regressing out nuisance covariates, fALFF reflects the proportion of meaningful physiological signal within the total frequency band. Higher fALFF indicates a greater proportion of physiological activity and lower noise. Meaningful signals were typically defined within 0.01–0.08 Hz. ReHo analysis was performed without smoothing during preprocessing and reflects local signal consistency. After metric calculation, smoothing and normalization were performed in SPM12. Significant differences were defined at the voxel level as P < 0.05, FDR-corrected.

**Supplementary Figure Legends**

**Figure S1. The c-Fos expression profile after contextual fear conditioning.** (A) Timeline. After contextual fear conditioning (CFC), rats were perfused at 0.5 h, 1 h, and 1.5 h, and the number of c-Fos⁺ cells in the PrL was detected by immunofluorescence. (B) Representative images of c-Fos and DAPI immunostaining in layers 2–5a of the PrL. Scale bar = 100 μm. (C) Representative images of c-Fos and DAPI immunostaining in layers 5b–6 of the PrL. Scale bar = 100 μm. (D) Percentage of c-Fos⁺/DAPI⁺ cells in layers 2–5a (one-way ANOVA with post hoc test, *F*_(3, 12)_ = 39.420, P < 0.001 vs naive). Data are expressed as mean ± SEM. n = 4/group. (E) Percentage of c-Fos⁺/DAPI⁺ cells in layers 5b–6 (one-way ANOVA with post hoc test, *F*_(3, 12)_ = 34.563, P < 0.001 vs naive). Data are expressed as mean ± SEM. n = 4/group.

**Figure S2.** **The prelimbic but not the infralimbic cortex shows early and delayed waves of c-Fos expression after contextual fear memory training.** (A) c-Fos⁺ cells (green) in different mPFC/PrL layers at 18 h after contextual fear memory training. (B) Representative images of c-Fos⁺ cells and DAPI immunostaining in layers 2–5a of the PrL. (C) Percentage of c-Fos⁺/DAPI⁺ cells in layers 2–5a (one-way ANOVA with post hoc test, *F*_(8, 27)_ = 47.929, P < 0.01 vs naive). (D) Representative images of c-Fos⁺ cells and DAPI immunostaining in the IL. Scale bar = 100 μm. (E) Percentage of c-Fos⁺/DAPI⁺ cells in the IL (one-way ANOVA with post hoc test, *F*_(8, 27)_ = 33.075, P < 0.01 vs naive). Data are expressed as mean ± SEM. n = 4/group.

**Figure S3.** **The neurons recruited during the early and delayed phases of the PrL are largely excitatory neurons.** (A) Representative images of c-Fos/CaMKII/DAPI and c-Fos/GAD67/DAPI immunofluorescence in the PrL at 0.5 and 18 h after contextual fear conditioning. Scale bar = 20 μm. (B, D) Over 40% of recruited PrL neurons were excitatory at both 0.5 and 18 h post-training. (C, E) Fewer than 10% of recruited neurons were inhibitory at the same time points.

**Figure S4. c-Fos protein expression was upregulated during both early and delayed phases following contextual fear conditioning.** (A) Rats were euthanized at 0.5 h or 18 h after CFC. (B, C) c-Fos protein levels were significantly increased at both 0.5 h and 18 h compared with naive controls. One-way ANOVA followed by post hoc test: *F_（_*_2,15）_= 30.761, P < 0.001 vs. naive.

**Figure S5. Expression of c-Fos marker gene was upregulated during early and delayed activation following contextual fear conditioning.**

(A) Experimental timeline. Rats were perfused at 0.25 h or 17.25 h after contextual fear conditioning. (B) Representative images of c-Fos marker gene/DAPI using In situ hybridization in the PrL. Scale bar = 100 μm. (C) Percentage of c-Fos⁺ cells relative to DAPI⁺ cells in the PrL. One-way ANOVA followed by post hoc test: *F_（_*_2,8）_= 128.306, P < 0.001 vs. naive.

**Figure S6. Microinfusion of anisomycin at 0.5 h after contextual fear conditioning did not alter c-Fos expression measured at 18 h.** (A) Experimental timeline. Rats received bilateral microinfusion of vehicle or anisomycin into the PrL at 0.5 h after contextual fear conditioning, and were perfused 17.5 h later. (B) Representative images of c-Fos/DAPI immunofluorescence in the PrL. Scale bar = 100 μm. (C) Percentage of c-Fos⁺ cells relative to DAPI⁺ cells in the PrL. Unpaired t-test: P = 0.221. Data are presented as mean ± SEM. n = 3 rats per group.

**Figure S7.** **Infusion of an mTOR signaling inhibitor into the PrL during the delayed phase impairs remote fear memory expression.** (A) Experimental timeline. Rats received microinfusion of vehicle or rapamycin into the PrL at 0.5 or 18 h after contextual fear conditioning. (B) Microinfusion of rapamycin into the PrL at 0.5 h post‑training had no effect on recent or remote fear memory expression (two‑way ANOVA: *F*_(1, 22)_ = 0.451, P = 0.509). (C) Microinfusion of rapamycin at 18 h post‑training selectively impaired remote, but not recent, fear memory (two‑way ANOVA: *F*_(1,16)_ = 8.318, P = 0.011). Data are presented as mean ± SEM. *P < 0.05. n = 9–12 rats per group.

**Figure S8.** **Microinfusion of a protein synthesis inhibitor into the PrL during the delayed phase impairs remote auditory fear memory expression.** (A) Experimental timeline. Rats received vehicle or anisomycin into the PrL at 0.5 or 18 h after auditory fear conditioning (AFC). Recent and remote memory tests were performed in a novel context on days 2 and 14 using the same auditory cue as training. (B, C) Microinfusion of anisomycin at 0.5 h post-AFC had no effect on recent or remote auditory fear memory expression (two-way ANOVA: *F*_(1, 15)_ = 0.053, P = 0.822). (D, E) Microinfusion of anisomycin at 18 h post-AFC selectively impaired remote, but not recent, auditory fear memory (two-way ANOVA: *F*_(1, 17)_ = 10.007, P = 0.006). Data are presented as mean ± SEM. *P < 0.05. n = 8–11 rats per group.

**Figure S9.** **Neurons recruited during the early and delay phases belong to different neuronal ensembles.** (A) Experimental timeline for labeling mPFC neurons at approximately 0.5 h and detecting endogenous c-Fos expression via immunofluorescence at 18 h following CFC. (B) Representative fluorescence images of labeled neurons in the PrL. Home cage-recruited neurons were labeled in the home cage control group; early-recruited neurons were labeled in the CFC group, while delayed-recruited neurons were identified by c-Fos immunolabeling. Scale bar = 100 μm. (C) Percentage of mCherry⁺ or c-Fos⁺ cells relative to total DAPI⁺ cells in the PrL (CFC vs. home cage, t-test, P < 0.005). The proportion of mCherry⁺/c-Fos⁺ double-positive neurons did not significantly differ from the predicted chance level, calculated as the product of the mCherry⁺/DAPI⁺ and c-Fos⁺/DAPI⁺ fractions in both home cage and CFC groups. n = 3–4 rats per group.

**Figure S10. 4 Hz, but not 20 Hz, blue light stimulation of ChR2-mCherry-expressing neurons in the delayed activation phase of the PrL induces the expression of remote fear memory.** (A) Experimental timeline. mPFC neurons activated during delayed activation phase after CFC were tagged with ChR2-mCherry. 2 days or 14days after CFC, the rats received 4 Hz or 20 Hz photostimulation in a distinct context. (B) Representative images showing the positions of optical cannula tips and ChR2-mCherry+ mPFC neurons. (C-F) Summary plots showing average freezing time in the presence and absence of photostimulation in the ChR2 group (Data in C-F are expressed as mean ± SEM. n = 7-8 rats per group. *P ≤ 0.05, unpaired t-test).

**Figure S11.** **Enhanced remote memory recall induced by SKF38393 during the delayed phase is not attributable to fear memory generalization.** (A) Rats were implanted with guide cannulas targeting the PrL and allowed 7 days of recovery. They then underwent CFC. SKF38393 was microinfused into the PrL 18 h after CFC. Contextual fear memory tests were conducted in the training context on days 2 and 14. A generalization test was performed in a novel context on day 15. (B) Microinfusion of SKF38393 into the PrL 18 h post-CFC enhanced remote fear memory retention on day 14 (unpaired t-test: P = 0.035) but did not affect performance in the generalization test (unpaired t-test: P > 0.05). Data are presented as mean ± SEM. *P < 0.05. n = 7–8 rats per group.

**Figure S12.** **β-adrenergic signaling mediates early mPFC actions on recent fear memory.** (A) Rats received microinfusion of vehicle, SCH23390, SKF38393, or propranolol into the PrL 0.5 h after CFC. (B) Microinfusion of SCH23390 into the PrL 0.5 h post-CFC had no effect on recent or remote fear memory expression (two-way ANOVA with post hoc test: *F*_(1,17)_ = 0.028, P = 0.869). (C) Microinfusion of SKF38393 into the PrL 0.5 h post-CFC did not alter recent or remote fear memory expression (two-way ANOVA with post hoc test: *F*_(1,15)_ = 0.199, P = 0.662). (D) Microinfusion of propranolol into the PrL 0.5 h post-CFC selectively impaired recent, but not remote, fear memory (two-way ANOVA with post hoc test: *F*_(1,16)_ = 4.732, P = 0.045). Data are presented as mean ± SEM. *P < 0.05. n = 8–10 rats per group. (E) Experimental timeline for sEPSC recordings of PrL pyramidal neurons before and after propranolol application in naive rats and rats at 0.5 h after CFC. (F) Representative sEPSC traces from PrL pyramidal neurons in the naive group (pink: naive baseline; red: naive + propranolol) and 0.5 h group (light blue: 0.5 h baseline; dark blue: 0.5 h + propranolol), Scale bar: 20 pA (vertical), 1 s(horizontal). (G) Cumulative distribution of sEPSC inter-event intervals and corresponding dot plots (inset) revealed that propranolol significantly decreased sEPSC inter-event intervals in PrL pyramidal neurons at 0.5 h post-CFC (*F*_(1, 15)_ = 15.15, P = 0.0014). (H) Cumulative distribution of sEPSC amplitudes and corresponding dot plots (inset) showed that propranolol significantly reduced sEPSC amplitude in PrL pyramidal neurons at 0.5 h post-CFC (*F*_(1, 15)_ = 14.17, P = 0.0019). Data are presented as mean ± SEM. *P < 0.05. n = 8–9 neurons from 8–9 slices, 3 rats per group.

.

**Figure S13. Chemogenetic modulation of the VTA-PrL projection during the delayed phase correlates with neuronal activity in the PrL.** (A, B) Representative images of c-Fos/DAPI immunofluorescence in the PrL. Scale bar = 100 μm. (C) In vivo CNO administration in rats expressing DIO-M4-mCherry significantly decreased the percentage of c-Fos⁺ cells relative to total DAPI⁺ cells in the PrL, compared with control rats expressing DIO-mCherry (unpaired t-test: P < 0.05). (D) In vivo CNO administration in rats expressing DIO-M3-mCherry significantly increased the percentage of c-Fos⁺ cells relative to total DAPI⁺ cells in the PrL, compared with control rats expressing DIO-mCherry (unpaired t-test: P < 0.05). Data are presented as mean ± SEM. *P < 0.05. n = 3–4 rats per group.

**Figure S14. The sufficiency of VTA-PrL pathway for the persistence of remote memory does not due to fear memory generalization.** (A) Rats received chemogenetic activating virus infusions into the PrL and were allowed 21 days of viral expression. On day 21, rats underwent CFC, and CNO was administered intraperitoneally (i.p.) 17 h post-CFC. Memory tests were performed in the training context on days 2 and 14, with a generalization test in a novel context on day 15. (B) CNO administration 17 h post-CFC improved remote fear memory retention on day 14 (unpaired t-test: P = 0.017) but did not alter generalization test performance (unpaired t-test: P > 0.05). Data are presented as mean ± SEM. *P < 0.05. n = 7–8 rats per group.

**Figure S15.** **VTA-PrL projection during the early phase have no effect on the persistence of fear memory.** (A) Experimental schematic: Retro-AAV-hSyn-EGFP-Cre (Retro-EGFP-Cre) was infused into the PrL, and rAAV-EF1α-DIO-hM4Di-mCherry (DIO-M4-mCherry) or rAAV-EF1α-DIO-hM3Dq-mCherry (DIO-M3-mCherry) was infused into the VTA. Three weeks later, rats received intraperitoneal (i.p.) CNO injection immediately after contextual fear conditioning (CFC). Brains were harvested 60 min after the remote fear memory test. (B, E) (Left) Retro-EGFP-Cre expression at the PrL infusion site (scale bar = 1 mm). (Right) High-magnification view of Retro-EGFP-Cre expression in the PrL (green: Retro-EGFP-Cre; blue: DAPI; scale bar = 50 μm). (C, F) (Left) Co-expression of Retro-EGFP-Cre and DIO-M4/M3-mCherry (DIO-mCherry for controls) in the VTA. (Right) High-magnification view of viral expression in the VTA (green: Retro-EGFP-Cre; red: DIO-M4/M3-mCherry or DIO-mCherry; blue: DAPI; scale bar = 50 μm). (D) Immediate CNO administration after CFC had no effect on recent or remote fear memory expression (two-way ANOVA with post hoc test: *F*_(1, 18)_ = 0.204, P = 0.657). (G) Immediate CNO administration after CFC did not alter recent or remote fear memory expression (two-way ANOVA with post hoc test: *F*_(1, 14)_ = 0.106, P = 0.750). Data are presented as mean ± SEM. n = 8–9 rats per group.

**Figure S16.** **Chemogenetic or pharmacological manipulation of the VTA-PrL projection during the delayed phase does not alter locomotor activity.** (A) Representative traces of locomotor activity. (B) Inhibition of the VTA–PrL projection during the delayed phase did not significantly alter total distance traveled (unpaired t-test: P > 0.05). (C) Inhibition of the VTA-PrL during the delayed phase did not significantly alter center zone time (unpaired t-test: P > 0.05). (D) Representative traces of locomotor activity. (E) Activation of the VTA-PrL during the delayed phase did not significantly alter total distance traveled (unpaired t-test: P > 0.05). (F) Activation of the VTA-PrL during the delayed phase did not significantly alter center zone time (unpaired t-test: P > 0.05). (G, J) Representative traces of locomotor activity. (H, K) Chemogenetic and pharmacological manipulation during the delayed phase did not significantly alter total distance traveled (unpaired t-test: P > 0.05). (I, L) Chemogenetic and pharmacological manipulation during the delayed phase did not significantly alter center zone time (unpaired t-test: P > 0.05). Data are presented as mean ± SEM. n = 5–7 rats per group.

**Figure S17.** **Subjective ratings among groups.** (A) Subjective ratings after fear learning in the 0.5 h and 9 h groups. Participants showed significantly higher fear scores toward CS+ than CS- (*t*_(77)_ = 23.28, P < 0.01). (B) Subjective ratings after fear learning in the 18 h and 36 h groups. Participants showed significantly higher fear scores toward CS+ than CS- (*t*_(84)_ = 18.30, P < 0.01). (C) Comparison of subjective rating ratios between fear learning and recent memory recall tests in the 0.5 h and 9 h groups. Repeated-measures ANOVA was performed on subjective rating ratios across four groups, with intervention time window (0.5 h vs. 9 h) and intervention type (cTBS vs. sham) as between-group factors, and experimental phase (learning phase vs. recent recall test) as the within-subject factor. Results showed no significant main effects of intervention time window (*F*_(1, 17)_ = 1.53, P = 0.23) or intervention type (*F*_(1, 17)_ = 0.12, P = 0.74), a significant main effect of experimental phase (*F*_(1, 17)_ = 18.99, P < 0.01), and no significant interaction effect (*F*_(1, 17)_ = 0.39, P = 0.54). In the recent recall test, fear scores were significantly lower in the 0.5 h cTBS group (0.5 h cTBS < 0.5 h sham, P = 0.03; 0.5 h cTBS < 9 h cTBS, P = 0.03; 0.5 h cTBS < 9 h sham, P < 0.01). (D) Comparison of subjective rating ratios between fear learning and remote memory recall tests in the 18 h and 36 h groups. Repeated-measures ANOVA was performed on subjective rating ratios across four groups, with intervention time window (18 h vs. 36 h) as the between-group factor, intervention type (cTBS vs. sham) as the between-group factor, and experimental phase (learning phase vs. remote recall test) as the within-subject factor. Results showed no significant main effects of intervention time window (*F*_(1, 15)_ = 0.00, P = 0.97) or intervention type (*F*_(1, 15)_ = 0.93, P = 0.35), a significant main effect of experimental phase (*F*_(1, 15)_ = 15.83, P < 0.01), and a significant interaction effect (*F*_(1, 15)_ = 10.48, P < 0.01). In the remote recall test, fear scores were lower in the 18 h cTBS group (18 h cTBS < 18 h sham, P = 0.02; 18 h cTBS < 36 h cTBS, P = 0.08; 18 h cTBS < 36 h sham, P = 0.15). *P < 0.05, **P < 0.01, ***P < 0.001. Data are presented as mean ± SEM.

**Figure S18.** **SCR trends during recent memory recall tests.** (A) Recent memory recall trends in the 0.5 h group. Repeated-measures ANOVA was performed on standardized differential SCR across five sessions of the recent fear memory test in participants from the 0.5 h and 9 h groups, with intervention time window (0.5 h vs. 9 h) and intervention type (cTBS vs. sham) as between-group factors, and test session as the within-subject factor (analyzed across sessions 1–5). Results showed significant main effects of intervention type (*F*_(1, 17)_ = 4.51, P = 0.05), no significant main effect of test session (*F*_(4, 14)_ = 0.51, P = 0.73), no significant main effects of intervention time window (*F*_(1, 17)_ = 0.08, P = 0.78) and no significant interaction effect (*F*_(4, 14)_ = 0.38, P = 0.82). Different intervention types produced distinct trends in recent fear memory changes. (B) Recent memory trends in the 9 h group. (C) Recent memory trends in the 18 h group. Repeated-measures ANOVA was performed on standardized differential SCR across five sessions of the recent fear memory test in participants from the 18 h and 36 h groups, with intervention time window (18 h vs. 36 h) and intervention type (cTBS vs. sham) as between-group factors, and test session as the within-subject factor (analyzed across sessions 1–5). Results showed no significant main effects of intervention time window (*F*_(1, 19)_ = 0.20, P = 0.66) or intervention type (*F*_(1, 19)_ = 0.00, P = 0.97), a significant main effect of test session (*F*_(4, 16)_ = 6.40, P < 0.01), and no significant interaction effect (*F*_(4, 16)_ = 1.43, P = 0.27). Post hoc analysis showed that standardized differential SCR in session 5 was significantly lower than that in session 1 (*F*_(4, 16)_ = 6.48, P < 0.01; session 5 vs. session 1: P < 0.01). Fear responses gradually decreased during the recent memory test. (D) Recent memory trends in the 36 h group. *P < 0.05, **P < 0.01, ***P < 0.001. Data are presented as mean ± SEM.

**Figure S19. Comparison of the last session of the recent memory recall test with the first session of the remote memory recall test.** (A) Comparison of the last session (session 5) of the recent memory test with the first session of the remote memory test in the 0.5 h group. Given that participants did not receive electric stimulation upon CS+ during the recent memory test, the test may have induced fear memory extinction. To further verify the effect of mPFC cTBS intervention on remote memory expression, repeated-measures ANOVA was performed on standardized differential SCR in session 5 of the recent memory test and the first session of the remote memory test. For the Spontaneous Recovery (SR) test, intervention time window (0.5 h vs. 9 h) and intervention type (cTBS vs. sham) were used as between-group factors, and test session (session 5 of recent memory test vs. first session of remote memory test) as the within-subject factor. Results showed no significant main effects of intervention time window (*F*_(1, 12)_ = 2.93, P = 0.11) or intervention type (*F*_(1, 12)_ = 0.28, P = 0.61), a significant main effect of test session (*F*_(1, 12)_ = 17.46, P < 0.01), and no significant interaction effect (*F*_(1, 12)_ = 0.21, P = 0.66). Fear responses in session 5 of the recent memory test were significantly lower than those in the first session of the remote memory test (P < 0.01). For the Reinstatement test, the same between-group (intervention time window: 0.5 h vs. 9 h; intervention type: cTBS vs. sham) and within-subject (test session) factors were used. Results showed no significant main effects of intervention time window (*F*_(1, 12)_ = 0.65, P = 0.44) or intervention type (*F*_(1, 12)_ = 0.58, P = 0.46), a significant main effect of test session (*F*_(1, 12)_ = 13.99, P < 0.01), and no significant interaction effect (*F*_(1, 12)_ = 0.04, P = 0.85). Fear responses in session 5 of the recent memory test were significantly lower than those in the first session of the remote memory test (P < 0.01). Participants still exhibited significant fear responses in the first session of the remote memory test, indicating that mPFC cTBS at 0.5 h after fear learning had no effect on remote fear memory. (B) Comparison of the last session of the recent memory test with the first session of the remote memory test in the 9 h group. (C) Comparison of the last session of the recent memory test with the first session of the remote memory test in the 18 h group. Repeated-measures ANOVA on standardized differential SCR in session 5 of the recent memory test and the first session of the remote memory test revealed significantly reduced fear responses in the remote memory test. For the SR test, intervention time window (18 h vs. 36 h) and intervention type (cTBS vs. sham) were used as between-group factors, and test session (session 5 of recent memory test vs. first session of remote memory test) as the within-subject factor. Results showed no significant main effects of intervention time window (*F*_(1, 17)_ = 1.81, P = 0.20) or intervention type (*F*_(1, 17)_ = 0.01, P = 0.93), a significant main effect of test session (*F*_(1, 17)_ = 11.30, P < 0.01), no significant overall interaction effect (*F*_(1, 17)_ = 0.91, P = 0.35), but a significant interaction effect between test session and intervention type (*F*_(1, 17)_ = 5.94, P = 0.03). Fear responses in session 5 of the recent memory test were significantly lower than those in the first session of the remote memory test (P < 0.01). For the Reinstatement test, intervention time window (18 h vs. 36 h) and intervention type (cTBS vs. sham) were used as between-group factors, and test session as the within-subject factor. Results showed no significant main effects of intervention time window (*F*_(1, 17)_ = 1.47, P = 0.24) or intervention type (*F*_(1, 17)_ = 0.94, P = 0.35), a significant main effect of test session (*F*_(1, 17)_ = 28.23, P < 0.01), no significant overall interaction effect (*F*_(1, 17)_ = 2.68, P = 0.12), but a significant interaction effect between test session and intervention type (*F*_(1, 17)_ = 10.46, P < 0.01). Fear responses in session 5 of the recent memory test were significantly lower than those in the first session of the remote memory test (P < 0.01). Participants still exhibited significantly higher fear responses in the first session of the remote memory test, but mPFC cTBS at 18 h after fear learning tended to reduce fear responses in the remote memory test. (D) Comparison of the last session of the recent memory test with the first session of the remote memory test in the 36 h group. *P < 0.05, **P < 0.01, ***P < 0.001: comparisons between session 5 of the recent memory test and the first session of the remote memory test in the cTBS group. ^#^P < 0.05, ^##^P < 0.01, ^###^P < 0.001: comparisons between session 5 of the recent memory test and the first session of the remote memory test in the sham group. Data are presented as mean ± SEM.

**Figure S20. Neural responses in different time windows.** (A) Functional connectivity (FC) between dmPFC and vmPFC during fear learning was significantly stronger for CS+ than CS− (*F*_(2, 85)_ = 6.5, P < 0.001). (B) FC between dmPFC and vmPFC was significantly higher in the 0.5 h group than in the 9 h group (P < 0.001). (C) FC between dmPFC and vmPFC was significantly higher in the 18 h group than in the 36 h group (P < 0.001). *P < 0.05, **P < 0.01, ***P < 0.001. Data are presented as mean ± SEM.

**Supplementary Table S1.** Demographic information of participants.

|  | **Participants** | **0.5 h group** | **9 h group** | **18 h group** | **36 h group** | ***χ^2^*/*F*** | ***p*** |
| --- | --- | --- | --- | --- | --- | --- | --- |
| Gender  (female) | 87 (44) | 23 (12) | 12 (5) | 31 (13) | 21 (14) | 3.51 | 0.32 |
| Age | 22.48 ± 0.33 | 23.61 ± 0.87 | 22.00 ± 0.58 | 22.19 ± 0.48 | 21.95 ± 0.57 | 1.45 | 0.24 |
| Education | 16.23 ± 0.25 | 16.65 ± 0.62 | 16.67 ± 0.69 | 15.83 ± 0.40 | 15.95 ± 0.39 | 0.63 | 0.60 |
| BMI | 22.25 ± 0.29 | 22.46 ± 0.53 | 20.73 ± 0.62 | 22.30 ± 0.50 | 22.80 ± 0.59 | 1.73 | 0.17 |
| SDS | 39.65 ± 1.03 | 36.05± 1.72 | 42.27 ± 2.40 | 39.27 ± 2.04 | 42.36 ± 1.86 | 2.08 | 0.11 |
| SAS | 34.95 ± 0.84 | 31.58 ± 1.39 | 36.82 ± 1.87 | 35.89 ± 1.65 | 36.11 ± 1.58 | 2.05 | 0.12 |
| PSQI | 4.49 ± 0.26 | 4.00 ± 0.51 | 4.73 ± 0.54 | 4.30 ± 0.49 | 5.11 ± 0.49 | 0.92 | 0.44 |
| MOCA | 28.43 ± 0.12 | 28.47 ± 0.21 | 28.55 ± 0.31 | 28.04 ± 0.24 | 28.83 ± 0.19 | 2.27 | 0.09 |
| Digit Span | 17.37 ± 0.29 | 16.00 ± 0.53 | 17.70 ± 0.75 | 17.87 ± 0.49 | 17.78 ± 0.53 | 2.57 | 0.06 |
| Maximum tolerance | 58.41 ± 1.43 | 57.38 ± 2.15 | 60.26 ± 2.56 | 58.22 ± 3.00 | 58.75 ± 3.02 | 0.13 | 0.94 |

**Supplementary Table S2.** Demographic information in 0.5h and 9h groups.

|  | **Participants** | **0.5 h group** | | **9 h group** | | ***χ^2^*/*F*** | ***P*** |
| --- | --- | --- | --- | --- | --- | --- | --- |
|  |  | *cTBS group* | *Sham group* | *cTBS group* | *Sham group* |  |  |
| Gender (female) | 78 (59) | 20 (15) | 18 (13) | 20 (16) | 20 (15) | 0.33 | 0.95 |
| Age | 24.24 ± 0.49 | 25.55 ± 1.10 | 22.83 ± 0.86 | 24.60 ± 0.73 | 23.85 ± 1.11 | 1.37 | 0.26 |
| Education | 17.50 ± 0.29 | 18.00 ± 0.47 | 16.83 ± 0.56 | 18.10 ± 0.58 | 17.00 ± 0.66 | 1.32 | 0.28 |
| BMI | 20.98 ± 0.28 | 21.43 ± 0.43 | 21.01 ± 0.63 | 20.84 ± 0.68 | 20.66 ± 0.47 | 0.36 | 0.78 |
| SDS | 36.61 ± 0.84 | 32.96 ± 1.55 | 38.19 ± 1.57 | 36.75 ± 1.46 | 38.50 ± 1.88 | 2.41 | 0.07 |
| SAS | 33.50 ± 0.76 | 32.63 ± 0.98 | 36.90 ± 1.77 | 32.25 ± 1.24 | 32.50 ± 1.80 | 2.15 | 0.10 |
| PSQI | 3.69 ± 0.21 | 3.84 ± 0.31 | 3.94 ± 0.48 | 3.50 ± 0.44 | 3.50 ± 0.48 | 0.28 | 0.84 |
| MOCA | 28.33 ± 0.13 | 28.36 ± 0.17 | 28.77 ± 0.22 | 27.89 ± 0.28 | 28.35 ± 0.25 | 2.19 | 0.10 |
| Digit Span | 16.55 ± 0.32 | 15.64 ± 0.80 | 17.24 ± 0.59 | 16.28 ± 0.55 | 16.85 ± 0.63 | 1.08 | 0.36 |
| Maximum tolerance | 82.26 ± 1.38 | 80.72 ± 2.81 | 80.14 ± 2.47 | 86.20 ± 2.53 | 79.94 ± 3.04 | 2.12 | 0.12 |
| AMT | 19.79% ± 0.01 | 19.35% ± 0.02 | 21.89% ± 0.02 | 17.60% ± 0.02 | 20.55% ± 0.01 | 1.42 | 0.24 |

**Supplementary Table S3.** Demographic information in 18h and 36h groups.

|  | **Participants** | **18 h group** | | **36 h group** | | ***χ^2^*/*F*** | ***P*** |
| --- | --- | --- | --- | --- | --- | --- | --- |
|  |  | *cTBS group* | *Sham group* | *cTBS group* | *Sham group* |  |  |
| Gender  (female) | 85 (55) | 22 (16) | 22 (11) | 21 (12) | 20 (16) | 0.01 | 0.94 |
| Age | 23.68 ± 0.25 | 24.41 ± 0.38 | 23.19 ± 0.38 | 24.00 ± 0.38 | 23.05 ± 0.77 | 1.75 | 0.16 |
| Education | 17.38 ± 0.26 | 17.55 ± 0.44 | 17.19 ± 0.31 | 17.68 ± 0.52 | 17.05 ± 0.78 | 0.31 | 0.82 |
| BMI | 22.50 ± 0.64 | 21.07 ± 0.52 | 24.02 ± 1.51 | 23.24 ± 1.52 | 21.68 ± 1.31 | 1.15 | 0.33 |
| SDS | 37.31 ± 0.75 | 36.93 ± 1.29 | 38.25 ± 1.69 | 35.06 ± 1.34 | 39.25 ± 1.63 | 1.50 | 0.22 |
| SAS | 34.55 ± 0.62 | 35.51 ± 1.13 | 34.06 ± 1.23 | 32.56 ± 1.12 | 36.19 ± 1.40 | 1.78 | 0.16 |
| PSQI | 4.20 ± 0.24 | 4.27 ± 0.40 | 4.45 ± 0.51 | 3.91 ± 0.49 | 4.20 ± 0.57 | 0.21 | 0.89 |
| MOCA | 27.89 ± 0.14 | 27.73 ± 0.30 | 27.89 ± 0.30 | 28.14 ± 0.19 | 27.79 ± 0.30 | 0.46 | 0.71 |
| Digit Span | 15.53 ± 0.26 | 15.64 ± 0.45 | 15.94 ± 0.53 | 15.14 ± 0.50 | 15.42 ± 0.63 | 0.40 | 0.75 |
| Maximum tolerance | 85.91 ± 1.30 | 86.93 ± 2.68 | 81.92 ± 3.13 | 86.23 ± 2.41 | 88.64 ± 2.00 | 1.18 | 0.32 |
| AMT | 16.22% ± 0.01 | 16.23% ± 0.01 | 20.57% ± 0.02 | 13.77% ± 0.01 | 14.35% ± 0.02 | 2.26 | 0.14 |

**Supplementary Table S4.** Summary of statistical analysis of animal study.

| **Fig.** | ***n*** | **Statistical test** | **Main effect 1** | **Main effect 2** | **Interaction** |
| --- | --- | --- | --- | --- | --- |
| Fig. 1C | *n* = 4/group | one-way ANOVA with *post hoc* test | Group  (*F*_8,27_ = 47.701, P < 0.001) |  |  |
| Fig. 1F | *n* = 14-19 neurons, 7-9 slices from 3 rats/group | one-way ANOVA with *post hoc* test, and a priori planned comparisons using unpaired t-tests | Group  (*F*_4,77_ = 5.697, P < 0.001);  0.5 h vs. Naive: t_29_ = -3.70, P = 0.001; 18 h vs. Naive: t_31_ = -2.138, P = 0.041 |  |  |
| Fig. 1G | *n* = 14-19 neurons, 7-9 slices from 3 rats/group | one-way ANOVA with *post hoc* test, and a priori planned comparisons using unpaired t-tests | Group  (*F*_4,77_ = 18.46, P < 0.0001);  0.5 h vs. Naive: t_29_ = -8.389, P < 0.001; 18 h vs. Naive: t_31_ = -7.027, P < 0.001 |  |  |
| Fig. 1I | Vehicle = 10  Anisomycin = 11 | two-way ANOVA with *post hoc* test | Group  (*F*_1,19_ = 1.638, P = 0.216) | Test day  (*F*_1,19_ = 20.570, P < 0.001) | Group × Test day  (*F*_1,19_ = 4.540, P = 0.046) |
| Fig. 1J | Vehicle = 9  Anisomycin = 9 | two-way ANOVA with *post hoc* test | Group  (*F*_1,16_ = 0.059, P = 0.810) | Test day  (*F*_1,16_ = 22.515, P *<* 0.001) | Group × Test day  (*F*_1,16_ = 0.649, P = 0.432) |
| Fig. 1K | Vehicle = 10  Anisomycin = 11 | two-way ANOVA with *post hoc* test | Group  (*F*_1,19_ = 5.821, P = 0.026) | Test day  (*F*_1,19_ = 48.317, P *<* 0.001) | Group × Test day  (*F*_1,19_ = 9.810, P = 0.005) |
| Fig. 1L | Vehicle = 10  Anisomycin = 9 | two-way ANOVA with *post hoc* test | Group  (*F*_1,17_ = 0.246, P = 0.626) | Test day  (*F*_1,17_ = 11.986, P = 0.003) | Group × Test day  (*F*_1,17_ = 0.029, P = 0.867) |
| Fig. 2C | *n* = 3/group | unpaired *t*-test | 0.5 h HC vs. 0.5 h CFC: t_4_ = 16.601, P < 0.001;  18 h HC vs. 18 h CFC: t_4_ = 7.562, P = 0.0016 |  |  |
| Fig. 2F | *n* = 3/group | two-way ANOVA with *post hoc* test | Group  (*F*_1,8_= 54.681, P = 0.002) | Test day  (*F*_1,8_ 42.231, P = 0.003) | Group × Test day  (*F*_1,8_= 30.208, P = 0.005) |
| Fig. 2I | *n* = 8-9/group | unpaired *t*-test | t_14_ = 0.106, P = 0.9173 |  |  |
| Fig. 2J | *n* = 8-9/group | unpaired *t*-test | t_14_ = 4.301, P = 0.0007 |  |  |
| Fig. 2K | *n* = 7-8/group | unpaired *t*-test | t_14_ = −0.777, P = 0.450 |  |  |
| Fig. 2L | *n =* 8-9/group | unpaired *t*-test | t_15_ = −10.018, P < 0.001 |  |  |
| Fig. 3B | Vehicle = 10  SCH = 10 | two-way ANOVA with *post hoc* test | Group  (*F*_1,18_ = 14.787, P = 0.001 | Test day  (*F*_1,18_ = 100.833, P *<* 0.001) | Group × Test day  (*F*_1,18_ = 5.026, P = 0.038) |
| Fig. 3C | Vehicle = 9  SKF = 9 | unpaired *t*-test | 2 day Vehicle vs. 2 day SKF: t_16_ = −0.244, P = 0.810;  14 day Vehicle vs. 14 day SKF: t_16_ = −2.222, P = 0.041 |  |  |
| Fig. 3D | Vehicle = 8  Propranolol = 8 | two-way ANOVA with *post hoc* test | Group  (*F*_1,14_= 3.134, P = 0.098) | Test day  (*F*_1,14_ = 2.840, P = 0.114) | Group × Test day  (*F*_1,14_= 0.038, P = 0.848) |
| Fig. 3G | *n* = 8-10 neurons, 8-10 slices from 3 rats/group | two-way ANOVA with *post hoc* test, and a priori planned comparisons using paired *t*-tests | Group × Test day  (*F*_1,19_ = 12.48, P = 0.0022);  Naive baseline vs. Naive + SCH: t_10_ = 0.076, P = 0.9407;  18 h baseline vs. 18 h + SCH: t_9_ = 3.642, P = 0.0054 |  |  |
| Fig. 3H | *n* = 8-10 neurons, 8-10 slices from 3 rats/group | paired *t*-test | Naive baseline vs. Naive + SCH: t_10_ = 0.401, P = 0.697;  18 h baseline vs. 18 h + SCH: t_9_ = 2.370, P = 0.042 |  |  |
| Fig. 3J | *n* = 8-10 neurons, 8-10 slices from 3 rats/group | paired *t*-test | Naive baseline vs. Naive + SKF: t_7_ = 8.11, P < 0.001;  18 h baseline vs. 18 h + SKF: t_11_ =2.40, P = 0.036 |  |  |
| Fig. 3K | *n* = 8-10 neurons, 8-10 slices from 3 rats/group | paired *t*-test | Naive baseline vs. Naive + SKF: t_7_ = - 2.5338, P = 0.0390;  18 h baseline vs. 18 h + SKF: t_11_ = - 4.0023, P = 0.0021 |  |  |
| Fig. 4F | CNO = 9  hM4Di CNO = 9 | two-way ANOVA with *post hoc* test | Group  (*F*_1, 16_ = 5.240, P = 0.036) | Test day  (*F*_1, 16_ = 51.817, P *<* 0.001) | Group × Test day  (*F*_1, 16_ = 10.473, P = 0.005) |
| Fig. 4I | CNO = 8  hM3Dq CNO = 8 | unpaired *t*-test | 2 day CNO vs. hM3Dq: t_14_ = 0.016, P = 0.987;  14 day CNO vs. hM3Dq: t_14_ = -2.65, P = 0.019 |  |  |
| Fig. 4M | hM4Di CNO+ Vehicle = 10  hM4Di CNO+SKF = 11 | two-way ANOVA with *post hoc* test | Group  (*F*_1, 19_ = 5.387, P = 0.032) | Test day  (*F*_1, 19_ = 87.671, P *<* 0.001) | Group × Test day  (*F*_1, 19_= 9.711, P = 0.006) |
| Fig. 4P | hM3Dq CNO+ Vehicle = 10  hM3Dq CNO+SCH = 8 | unpaired *t*-test | 2 day Vehicle vs. SCH: t_16_ = 0.021, P = 0.983;  14 day Vehicle vs. SCH: t_16_ = 3.643, P = 0.0022 |  |  |
| Fig. S1D | *n* = 4/group | one-way ANOVA with *post hoc* test | Group  *F*_3, 12_ *=* 39.420*,*  P < 0.001 |  |  |
| Fig. S1E | *n* = 4/group | one-way ANOVA with *post hoc* test | Group  *F*_3, 12_ *=* 34.563*,* P < 0.001 |  |  |
| Fig. S2C | *n* = 4/group | one-way ANOVA with *post hoc* test | Group  (*F*_8, 27_ = 47.929, P *<* 0.001) |  |  |
| Fig. S2E | *n* = 4/group | one-way ANOVA with *post hoc* test | Group  (*F*_8, 27_ = 33.075, *P <* 0.001) |  |  |
| Fig. S4C | *n =* 6/group | one-way ANOVA with *post hoc* test | Group  (*F*_2, 15_ = 30.761, *P <* 0.001) |  |  |
| Fig. S5C | *n =* 3/group | one-way ANOVA with *post hoc* test | (*F*_2,_ _6_ 128.306, *P* *<* 0.001) |  |  |
| Fig. S6C | *n* = 3/group | unpaired *t*-test | t_4_ = -1.45, P = 0.221 |  |  |
| Fig. S7B | Vehicle = 12  Rapamycin = 12 | two-way ANOVA with *post hoc* test | Group  (*F*_1, 22_ = 0.03, P = 0.864) | Test day  (*F*_1, 22_ = 47.161, P *<* 0.001) | Group × Test day  (*F*_1, 22_ = 0.451, P = 0.509) |
| Fig. S7C | Vehicle = 9  Rapamycin = 9 | two-way ANOVA with *post hoc* test | Group  (*F*_1, 16_ = 7.253, P = 0.016) | Test day  (*F*_1, 16_ = 25.416, P *<* 0.001) | Group × Test day  (*F*_1, 16_ = 8.318, P = 0.011) |
| Fig. S8C | Vehicle = 8  Anisomycin = 9 | two-way ANOVA with *post hoc* test | Group  (*F*_1, 15_ = 0.023, P = 0.822) | Test day  (*F*_1, 15_ = 23.948, P *<* 0.001) | Group × Test day  (*F*_1, 15_ = 0.053, P = 0.822) |
| Fig. S8E | Vehicle = 10  Anisomycin = 11 | two-way ANOVA with *post hoc* test | Group  (*F*_1,17_ = 7.929, P = 0.012) | Test day  (*F*_1,17_ = 23.173, P *<* 0.001) | Group × Test day  (*F*_1,17_ = 10.007, P = 0.006) |
| Fig. S9C | n = 3–4/group | unpaired *t*-test | 0.5 h HC vs. 0.5 h CFC: t_5_ = 13.70, P < 0.0001;  18 h HC vs. 18 h CFC: t_5_ = 32.13, P < 0.0001 |  |  |
| Fig. S10C | n =8/group | paired *t*-test | Laser off vs. Laser on: t_7_= -0.059, P = 0.954 |  |  |
| Fig. S10D | n =8/group | paired *t*-test | Laser off vs. Laser on: t_7_= -6.930, P *<* 0.001 |  |  |
| Fig. S10E | n =7/group | paired *t*-test | Laser off vs. Laser on: t_6_= -0.955, P = 0.376 |  |  |
| Fig.  S10F | n =7/group | paired *t*-test | Laser off vs. Laser on: t_6=_ -1.459, P = 0.195 |  |  |
| Fig. S11B | Vehicle = 8  SKF = 8 | unpaired *t*-test | 2 day Vehicle vs. SKF: t_14_ = 0.194, P = 0.8489;  14 day Vehicle vs. SKF: t_10_ = 3.66, P = 0.004  15 day Vehicle vs. SKF: t_14_ = 0.818, P = 0.426 |  |  |
| Fig. S12B | Vehicle = 10  SCH = 10 | two-way ANOVA with *post hoc* test | Group  (*F*_1,17_ = 0.028, P = 0.869) | Test day  (*F*_1,17_ = 41.437, P < 0.001) | Group × Test day  (*F*_1,17_ = 0.352, P = 0.561) |
| Fig. S12C | Vehicle = 9  SKF = 8 | two-way ANOVA with *post hoc* test | Group  (*F*_1,15_ = 0.221, P = 0.645) | Test day  (*F*_1,15_ = 66.608, P < 0.001) | Group × Test day  (*F*_1,15_ = 0.199, P = 0.662) |
| Fig. S12D | Vehicle = 9  Propranolol = 9 | two-way ANOVA with *post hoc* test | Group  (*F*_1,16_ = 7.258, P = 0.016) | Test day  (*F*_1,16_ = 11.698, P = 0.004) | Group × Test day  (*F*_1,16_ = 4.732, P = 0.045) |
| Fig. S12G | *n* = 8-9 neurons, 8-9 slices from 3 rats/group | paired *t*-test | Naive baseline vs. Naive + Prop: t_8_ = −0.82, P = 0.432;  0.5 h baseline vs. 0.5 h + SKF: t_7_ = 6.91, P = 0.0002 |  |  |
| Fig. S12H | *n* = 8-9 neurons, 8-9 slices from 3 rats/group | paired *t*-test | Naive baseline vs. Naive + SKF: t_8_ = −0.40, P = 0.699;  18 h baseline vs. 18 h + SKF: t_7_ = 6.52, P < 0.0001 |  |  |
| Fig. S13C | CNO = 4  hM4Di CNO = 3 | unpaired *t*-test | t_6_ = 2.93, P = 0.027 |  |  |
| Fig. S13D | CNO = 4  hM4Di CNO = 4 | unpaired *t*-test | t_6_ = −4.53, P = 0.004 |  |  |
| Fig. S14B | hM3Dq Vehicle = 7;  hM3Dq CNO= 7 | unpaired *t*-test | 2 day Vehicle vs. CNO: t_12_ = 0.44, P = 0.667;  14 day Vehicle vs. CNO: t_12_ = -2.72, P = 0.019  15 day Vehicle vs. CNO: t_12_ = -0.49, P = 0.632 |  |  |
| Fig. S15D | CNO = 10  hM4Di CNO = 10 | two-way ANOVA with *post hoc* test | Group  (*F*_1, 18_ = 0.086, P = 0.773) | Test day  (*F*_1, 18_ = 18.095, P < 0.001) | Group × Test day  (*F*_1, 18_ = 0.204, P = 0.657) |
| Fig. S15G | CNO = 4  hM3Dq CNO = 4 | two-way ANOVA with *post hoc* test | Group  (*F*_1, 14_ = 0.127, P = 0.727) | Test day  (*F*_1, 14_ = 15.709, P = 0.001) | Group × Test day  (*F*_1, 14_ = 0.106, P = 0.750) |
| Fig. S16B | *n* = 5-7/group | unpaired *t*-test | t_9_ = −0.31, P = 0.768 |  |  |
| Fig. S16C | *n* = 5-7/group | unpaired *t*-test | t_12_ = 0.41, P = 0.689 |  |  |
| Fig. S16E | *n* = 5-7/group | unpaired *t*-test | t_12_ = 0.41, P = 0.689 |  |  |
| Fig. S16F | *n* = 5-7/group | unpaired *t*-test | t_12_ = 0.41, P = 0.689 |  |  |
| Fig. S16H | *n* = 5-7/group | unpaired *t*-test | t_10_ = 0.94, P = 0.368 |  |  |
| Fig. S16I | *n* = 5-7/group | unpaired *t*-test | t_10_ = 0.62, P = 0.547 |  |  |
| Fig. S16K | *n* = 5-7/group | unpaired *t*-test | t_10_ = −0.02, P = 0.981 |  |  |
| Fig. S16L | *n* = 5-7/group | unpaired *t*-test | t_10_ = −0.70, P = 0.499 |  |  |

**Supplementary Table S5.** Summary of statistical analysis of human study.

| **Figure** | ***n*** | **Statistical test** | **Main effect 1** | **Main effect 2** | **Time effect** | **Interaction** |
| --- | --- | --- | --- | --- | --- | --- |
| Fig. 5B | 0.5 h cTBS *n* = 20  0.5 h sham *n* = 18  9 h cTBS *n* = 20  9 h sham *n* = 20 | Repeated-measures ANOVA | Time window of intervention  (*F*_1,17_ = 0.05, P = 0.83) | Type of intervention  (*F*_1,17_ = 0.17, P = 0.68) | Session  (*F*_3,15_ = 29.81, P *<* 0.01) | *F*_3,15_ = 1.04, P = 0.41 |
| Fig. 5C | 18 h cTBS *n* = 22  18 h sham *n* = 22  36 h cTBS *n* = 21  36 h sham *n* = 20 | Repeated-measures ANOVA | Time window of intervention  (*F*_1,19_ = 3.62, P = 0.07) | Type of intervention  (*F*_1,19_ = 0.34, P = 0.57) | Session  (*F*_3,17_ = 23.88, P *<* 0.01) | *F*_3,17_ = 1.53, P = 0.24 |
| Fig. 5D | 0.5 h cTBS *n* = 20  0.5 h sham *n* = 18  9 h cTBS *n* = 20  9 h sham *n* = 20 | One-way ANOVA | *F*_3,73_ = 3.41, P = 0.02 |  |  |  |
| Fig. 5E | 18 h cTBS *n* = 22  18 h sham *n* = 22  36 h cTBS *n* = 21  36 h sham *n* = 20 | One-way ANOVA | *F*_3,81_ = 0.33, P = 0.80 |  |  |  |
| Fig. 5F | 0.5 h cTBS *n* = 20  0.5 h sham *n* = 18  9 h cTBS *n* = 20  9 h sham *n* = 20 | One-way ANOVA | *F*_3,69_ = 0.87, P = 0.46 |  |  |  |
| Fig. 5G | 18 h cTBS *n* = 22  18 h sham *n* = 22  36 h cTBS *n* = 21  36 h sham *n* = 20 | One-way ANOVA | *F*_3,79_ = 4.16, P = 0.01 |  |  |  |
| Fig. S17A | 0.5 h cTBS *n* = 20  0.5 h sham *n* = 18  9 h cTBS *n* = 20  9 h sham *n* = 20 | Unpaired *t* test  (CS+>CS-) | t_77_ = 23.28, P *<* 0.01 |  |  |  |
| Fig. S17B | 18 h cTBS *n* = 22  18 h sham *n* = 22  36 h cTBS *n* = 21  36 h sham *n* = 20 | Unpaired *t* test  (CS+>CS-) | t_84_ = 18.30, P *<* 0.01 |  |  |  |
| Fig. S17C | 0.5 h cTBS *n* = 20  0.5 h sham *n* = 18  9 h cTBS *n* = 20  9 h sham *n* = 20 | Repeated-measures ANOVA | Time window of intervention  (*F*_1,17_ = 1.53, P = 0.23) | Type of intervention  (*F*_1,17_ = 0.12, P = 0.74) | Session  (*F*_1,17_ = 18.99, P *<* 0.01) | *F*_1,17_ =0.39, P = 0.54 |
| Fig. S17D | 18 h cTBS *n* = 22  18 h sham *n* = 22  36 h cTBS *n* = 21  36 h sham *n* = 20 | Repeated-measures ANOVA | Time window of intervention  (*F*_1,15_= 0.00, P = 0.97) | Type of intervention  (*F*_1,15_ = 0.93, P = 0.35) | Session  (*F*_1,15_ = 15.83, P *<* 0.01) | *F*_1,15_ = 10.48, P *<* 0.01 |
| Fig. S18A, B | 0.5 h cTBS *n* = 20  0.5 h sham *n* = 18  9 h cTBS *n* = 20  9 h sham *n* = 20 | Repeated-measures ANOVA | Time window of intervention  (*F*_1,17_ = 0.06, P = 0.81) | Type of intervention  (*F*_1,17_ = 0.42, P = 0.53) | Session  (*F*_3,15_ = 3.72, P = 0.03) | *F*_3,15_ = 1.89, P = 0.17 |
| Fig. S18C, D | 18 h cTBS *n* = 22  18 h sham *n* = 22  36 h cTBS *n* = 21  36 h sham *n* = 20 | Repeated-measures ANOVA | Time window of intervention  (*F*_1,19_ = 0.21, P = 0.65) | Type of intervention  (*F*_1,19_ = 0.00, P = 0.98) | Session  (*F*_4,16_ = 6.48, P *<* 0.01) | *F*_4,16_ = 1.43, P = 0.27 |
| Fig. S19A, B | 0.5 h cTBS *n* = 18  0.5 h sham *n* = 18  9 h cTBS *n* = 18  9 h sham *n* = 19 | Repeated-measures ANOVA | Time window of intervention  SR test  (*F*_1,12_ = 2.93, P = 0.11)  Reinstatement test  (*F*_1,12_ = 0.65, P = 0.44) | Type of intervention  SR test  (*F*_1,12_ = 0.28, P = 0.61)  Reinstatement test  (*F*_1,12_ = 0.58, P = 0.46) | Session  SR test  (*F*_1,12_ = 17.46, P *<* 0.01)  Reinstatement test  (*F*_1,12_ = 13.99, P *<* 0.01) | SR test  *F*_1,12_ = 0.21, P = 0.66  Reinstatement test  *F*_1,12_ = 0.04, P = 0.85 |
| Fig. S19C, D | 18 h cTBS *n* = 22  18 h sham *n* = 21  36 h cTBS *n* = 21  36 h sham *n* = 19 | Repeated-measures ANOVA | Time window of intervention  SR test  (*F*_1,17_ =1.81, P = 0.20)  Reinstatement test  (*F*_1,17_ = 1.47, P = 0.24) | Type of intervention  SR test  (*F*_1,17_ = 0.01, P = 0.93)  Reinstatement test  (*F*_1,17_ = 0.94, P = 0.35) | Session  SR test  (*F*_1,17_ = 11.30, P *<* 0.01)  Reinstatement test  (*F*_1,17_ = 28.23, P *<* 0.01) | Session*Type  SR test  *F*_1,17_ = 5.94, P = 0.03  Reinstatement test  *F*_1,17_ = 10.46, P *<* 0.01 |

**Supplementary Table S6.** Activation and deactivation in different time windows.

Peak coordinates of the brain regions show differences at different phases at P_FDR_ < .05

| **Anatomical description** | **Nb of voxels** | **MNI coordinates** | | | ***F*** | **P*_FDR_*** |
| --- | --- | --- | --- | --- | --- | --- |
|  |  | **x** | **y** | **z** |  |  |
| **Fear memory task: CS+>CS-** | | | | | | |
| Medial Prefrontal Cortex | 578 | 33 | 51 | 3 | 5.51 | <0.001 |
| Anterior Cingulate Cortex | 456 | -12 | 18 | 27 | 4.89 | <0.001 |
| Inferior Prefrontal Cortex | 272 | -30 | 51 | -3 | 5.11 | <0.001 |
| Supramarginal Gyrus. | 489 | 38 | 8 | 64 | 5.24 | 0.002 |
| **Fear memory task-Functional connectivity: CS+>CS-** | | | | | | |
| Ventralmedial Prefrontal Cortex | 2736 | -5 | 52 | -10 | 5.51 | <0.001 |
| Lateral Occipital Cortex | 1844 | -48 | -72 | 26 | 4.99 | <0.001 |
| Lateral Occipital Cortex | 542 | 38 | -48 | 38 | 4.78 | <0.001 |
| Middle Frontal Gyrus | 489 | 38 | 8 | 64 | 4.75 | <0.001 |
| Middle Frontal Gyrus | 275 | 42 | 34 | 28 | 4.43 | <0.001 |
| Cerebellum 7b | 77 | -26 | -78 | -52 | 3.95 | <0.001 |
| **Before intervention: 0.5h>9h** | | | | | | |
| Ventral-medial Prefrontal Cortex | 41 | -6 | 51 | -6 | 8.81 | <0.001 |
| Supramarginal Gyrus | 31 | -57 | -63 | 33 | 5.02 | 0.004 |
| Middle Frontal Gyrus | 21 | -51 | 9 | 48 | 4.43 | 0.014 |
| Superior Frontal Gyrus | 21 | -24 | 36 | 51 | 4.03 | 0.014 |
| **Before intervention: 18h>36h** | | | | | | |
| Dorsal-medial Prefrontal Cortex | 38 | 0 | 33 | 39 | 5.06 | 0.002 |
| Ventral-medial Prefrontal Cortex | 12 | -9 | 60 | -15 | 4.21 | 0.029 |
| Superior Frontal Gyrus | 5 | -12 | 12 | 69 | 4.11 | 0.201 |
| Inferior Frontal Gyrus | 5 | -39 | 24 | 24 | 3.99 | 0.201 |
| **ALFF: Before intervention > After intervention** | | | | | | |
| Ventral-medial Prefrontal Cortex | 106 | 15 | 63 | 12 | -5.85 | <0.001 |
| Inferior Frontal Gyrus | 66 | -33 | 57 | 0 | -5.55 | <0.001 |
| **fALFF: Before intervention > After intervention** | | | | | | |
| Orbitofrontal Cortex | 51 | -6 | 39 | -12 | -5.72 | 0.01 |
| Dorsal-medial Prefrontal Cortex | 35 | -36 | 21 | 48 | -4.92 | 0.04 |
| **ReHo: Before intervention > After intervention** | | | | | | |
| Visual Association Cortex | 441 | 30 | -90 | 27 | -8.17 | <0.001 |
| Ventral-medial Prefrontal Cortex | 177 | -6 | 42 | -3 | -6.28 | <0.001 |
| Orbitofrontal Cortex | 81 | 15 | 66 | -9 | -5.29 | 0.03 |
